# Killer whale respiration rates

**DOI:** 10.1371/journal.pone.0302758

**Published:** 2024-05-15

**Authors:** Tess M. McRae, Beth L. Volpov, Evan Sidrow, Sarah M. E. Fortune, Marie Auger-Méthé, Nancy Heckman, Andrew W. Trites

**Affiliations:** 1 Institute for the Oceans and Fisheries, Marine Mammal Research Unit, University of British Columbia, Vancouver, BC, Canada; 2 Department of Statistics, University of British Columbia, Vancouver, BC, Canada; 3 Institute for the Oceans and Fisheries, University of British Columbia, Vancouver, BC, Canada; Animal Health Centre, CANADA

## Abstract

Measuring breathing rates is a means by which oxygen intake and metabolic rates can be estimated to determine food requirements and energy expenditure of killer whales (*Orcinus orca*) and other cetaceans. This relatively simple measure also allows the energetic consequences of environmental stressors to cetaceans to be understood but requires knowing respiration rates while they are engaged in different behaviours such as resting, travelling and foraging. We calculated respiration rates for different behavioural states of southern and northern resident killer whales using video from UAV drones and concurrent biologging data from animal-borne tags. Behavioural states of dive tracks were predicted using hierarchical hidden Markov models (HHMM) parameterized with time-depth data and with labeled tracks of drone-identified behavioural states (from drone footage that overlapped with the time-depth data). Dive tracks were sequences of dives and surface intervals lasting ≥ 10 minutes cumulative duration. We calculated respiration rates and estimated oxygen consumption rates for the predicted behavioural states of the tracks. We found that juvenile killer whales breathed at a higher rate when travelling (1.6 breaths min^-1^) compared to resting (1.2) and foraging (1.5)—and that adult males breathed at a higher rate when travelling (1.8) compared to both foraging (1.7) and resting (1.3). The juveniles in our study were estimated to consume 2.5–18.3 L O_2_ min^-1^ compared with 14.3–59.8 L O_2_ min^-1^ for adult males across all behaviours based on estimates of mass-specific tidal volume and oxygen extraction. Our findings confirm that killer whales take single breaths between dives and indicate that energy expenditure derived from respirations requires using sex, age, and behavioural-specific respiration rates. These findings can be applied to bioenergetics models on a behavioural-specific basis, and contribute towards obtaining better predictions of dive behaviours, energy expenditure and the food requirements of apex predators.

## Introduction

Cetaceans, like all mammals, consume oxygen as they breathe to sustain cellular respiration and provide energy needed for physiological functions and activities. As such, the metabolism and amount of energy animals expend can be determined from the amounts of oxygen they consume (L O_2_ min ^-1^)—which can be directly measured using respiratory calorimetry systems, or indirectly estimated from numbers of breaths taken [1]. Obtaining indirect estimates of oxygen consumption requires 1) monitoring respiratory rates (the number of breaths per minute), 2) calculating the tidal volume (the amount of air inhaled and exhaled during a single breath at rest), and 3) estimating the efficiency of gas exchanges in the lungs—all of which can be directly observed, measured, or estimated [1–3]. Counts of breaths (and associated changes in breathing rates that might occur over time) can also be used to guide conservation efforts to protect cetaceans and their habitats, as well as provide insights into the health of cetaceans and the impacts that human activities have on them.

Respiration rates are increasingly being used as an indirect means to calculate field metabolic rates, oxygen consumption and energy expenditure of cetaceans in the wild [4–6]. Breaths can be easily counted each time an animal surfaces [4,7,8], and can be averaged over a series of dives to calculate respiration rates [3]. Respiration rate can then be used to indirectly calculate oxygen consumption after making assumptions about the physiology of free-ranging cetaceans, which can ultimately be used to estimate prey requirements [2,3].

Killer whales (*Orcinus orca*) are thought to only take a single breath between dives. Previous studies have estimated oxygen consumption of free-ranging killer whales based on qualitative field observations [9] or acoustic recordings that captured respirations [10]. Assuming that killer whales only take single breaths between surface intervals means that counting surfacings from time-depth data is equivalent to counting breaths, which allows for surfacing intervals to be used to estimate energy expenditures. However, this has not been explicitly validated. Nor is it known whether the assumed single breath applies to all behavioural states (e.g., travelling, foraging, and resting).

Activity-specific metabolic rates are needed to parameterize bioenergetic models that estimate the differing amounts of energy cetaceans require to support the different activities they engage in each day. These can be obtained by knowing the volume of air inhaled and how much oxygen is exchanged per breath—as well as knowing the relationship between respiration rates and different behaviours such as travelling, foraging and resting. Oxygen exchange can be determined from trained animals [3], and activity budgets can be derived from boat-based focal follows [8] or from land-based tracking of individual whales [3–5]. However, categorizing behaviours of killer whales from boats and land—in the absence of subsurface data or observations—is prone to uncertainty related to unknown underwater behaviour.

One means to comprehensively identify cetacean behaviours and estimate concurrent respiration rates is to categorize the movements of cetaceans from data recorded by animal-borne biologging tags [11]. However, attempts to identify behaviours using kinematic variables, time-depth data, and Hidden Markov models (HMMs) have tended to categorize abstract activity states (e.g. State 1, 2 and 3) rather than biologically meaningful behavioural states [e.g., foraging, travelling and resting; 12–15]. Such descriptions and assertions of behavioural states derived from analysis of movements (e.g., HMMs) need validation, as well as clearly defined functions that are biologically meaningful to be useful for predicting energetic costs.

Many analyses have applied HMMs to cetacean data to predict behaviors that occurred during individual dives lasting a few minutes rather than describe behaviours that occur over longer sequences of dives lasting 10 minutes or more [12–16]. Unfortunately, counting breaths over short durations can result in inaccurate predictions of energy expenditure if animals have not had enough time to off-load CO_2_ and balance their O_2_ stores [17–19]. In the case of killer whales, respiration rates should be calculated over durations ≥ 10 minutes to avoid biased and inaccurate estimates of metabolic rates [3,4,8]. Fortunately, HHMMs (i.e., Hierarchical HHMMs) have been developed that can simultaneously categorize behaviours at multiple temporal scales [i.e., they can jointly define individual fine-scale dive types and coarse-scale behaviours of tracks comprised of several dives; 14,20].

We sought to determine the respiration rates of killer whales and how they differ by behavioural state by counting total breaths per surface interval observed on drone videos for different behavioural states occurring over durations ≥ 10 minutes. We then matched these drone video labels to time-depth data from animal-borne tags to inform an HHMM to predict whether tracks of dives were associated with resting, travelling, or foraging behavioural states. Output from the HHMM was then used to calculate behavioural-specific respiration rates, and estimate oxygen consumption for both adult male and juvenile resident killer whales. Our methodology to quantify killer whale behaviours, and our estimates of respiration rates can be applied to bioenergetics models on a sex, age, and behavioural-specific basis—and contribute to assessing the impacts of human activities on killer whales by providing a new method to accurately determine what whales are doing based solely on dive-depth data, and a better means to obtain estimates of energy expended by free-ranging killer whales under changing conditions.

## Materials and methods

### Data collection

We collected data from 11 northern and southern resident killer whales (*Orcinus orca*) in August 2020 off the coast of British Columbia in Queen Charlotte Sound, Queen Charlotte Strait, Johnstone Strait, and Juan de Fuca Strait (Table 1). Whale ID, sex, birth year and age as of 2020 were determined from catalogues of known individuals [21,22]. All 11 animals carried video cameras and time-depth dataloggers (CATS tags, Customizable Animal Tracking Solutions, www.cats.is), and 8 of the killer whales were simultaneously followed using an unmanned aerial vehicle (UAV) drone. We categorized whales into age classes with juveniles defined as 4–12 years, adult males > 13 years, and adult females as > 12 years with a recorded birth [7,23,24]. One of the females we tagged (R48) did not have a calf during tag deployment but was later identified as an adult female after a calf attributed to her was added to the catalogue. Based on her body mass and young age at the time of tagging, as well as preliminary statistical analysis, we included R48 in our juvenile category (See S2 Appendix for details). All killer whale data were collected under The University of British Columbia Animal Care Permit no. A19-0053 and Fisheries and Oceans Canada Marine Mammal Scientific License for Whale Research no. XMMS 6 2019.

**Table 1:** Summary of data collected on 11 resident killer whales.

Whale ID	Sex	Type	Birth year	Age	Age class	Tag date 2020	Total surface intervals on drone	Total surface intervals on CATS tag	CATS tag attachment duration (hours)
A113	Female	NRKW	2016	4	Juvenile	Aug. 22	264	872	7.4
R48	Female	NRKW	2006	14	Adult	Aug. 28	30	310	4.7
A100	Unknown	NRKW	2011	9	Juvenile	Aug. 20	4	356	3.6
R58	Unknown	NRKW	2011	9	Juvenile	Aug. 28	20	94	1.4
I145	Unknown	NRKW	2014	6	Juvenile	Aug. 30	12	461	5.1
D26	Unknown	NRKW	2010	10	Juvenile	Aug. 31	59	575	6.4
I129	Unknown	NRKW	2009	11	Juvenile	Aug. 30	No video	495	6.2
I107	Male	NRKW	2004	16	Adult	Aug. 25	66	1378	12.3
D21	Male	NRKW	2005	15	Adult	Aug. 31	21	1812	19.9
L87	Male	SRKW	1992	28	Adult	Sept. 10	No video	963	8.7
L88	Male	SRKW	1993	27	Adult	Sept. 10	No video	802	8.3
							**Total = 476**	**Total = 8118**	

Whale ID’s, sexes, birth years, ages, and age class [21–23] of nine northern (NRKW) and two southern resident (SRKW) killer whales equipped with biologging devices. Total individual dives on animal borne tags were 3163 for juveniles and 4955 for adult males. Also shown are the number of individual surface intervals (equivalent to the number of individual dives) recorded by the drone video and animal-borne tag for each whale, along with animal-borne tag attachment durations. Animals tagged on the same days did not have synchronous breathing and were treated independently.

#### Time-depth data collection

We tagged animals with suction cup CATS biologgers that were equipped with time-depth recorders, forward-facing underwater cameras, passive acoustic recorders (96 kHz), tri-axial accelerometers, magnetometers, gyroscopes, and satellite telemetry for asset retrieval. To deploy the animal-borne tags, a small vessel approached the focal group of killer whales and waited for a killer whale to surface near the vessel. Animal-borne tags were temporarily attached using suction cups and an adjustable 8m long carbon-fiber pole. Location of tag attachment varied, but preference was given near the base of the animal’s dorsal fin. The animal-borne tags continuously recorded dive movements for the duration of time the tag was attached. The animal-borne tags remained attached from 1.4 to 19.9 hours (Table 1). Following each tagging event, a focal follow was conducted until daylight was lost or the tag detached via galvanic release. During the follows, aerial drone flights (Inspire2) occurred to obtain video and still images of the tagged whale.

#### Behavioural states and respiration data observed from drones

We collected video data from a UAV (i.e., drone) for 8 of the 11 whales carrying the animal-borne tags. Once an individual was successfully tagged, we deployed a drone to follow the whale and take video footage at the surface. Due to battery limitations and the inability of the drone to capture video below the surface, the drone video data collected were not random subsamples of all dives recorded by the animal-borne tags and were biased towards shallower dives. We did collect drone video data on deeper and longer duration dives (up to 58 m), but these dive types did not have the same probability of being captured on the drone video as the shallower dives. This was due to the drone often losing track of the focal whale during deeper longer dives because of the difficulty of anticipating where the whale would surface.

**Fig 1:**
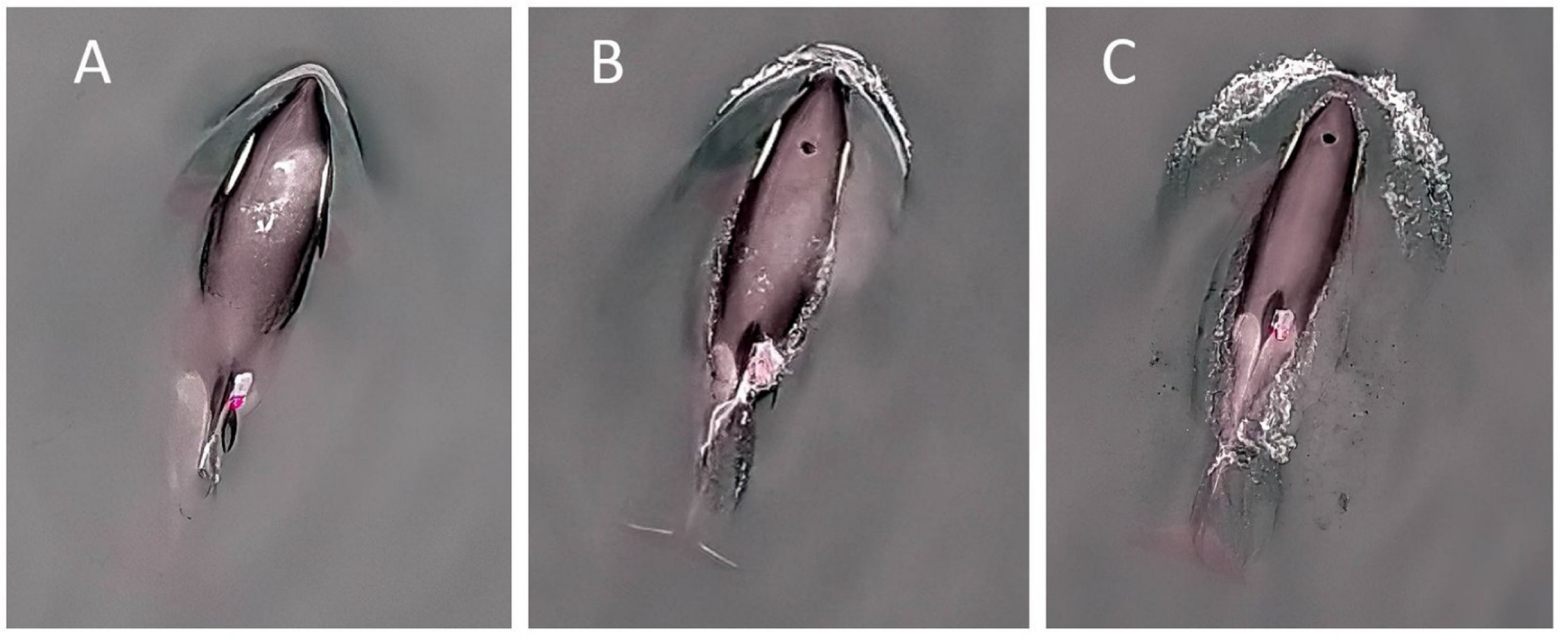
Still images from UAV drone video showing datalogger placement and respiration. Photographs showing the animal-borne datalogger attached with suction cups to a juvenile northern resident killer whale (A113). Views show (A) the whale’s breath immediately after surfacing, (B) mid-blow, and (C) after the blow has dissipated. Photo credit: Keith Holmes.

Behavioural states of the tagged whales were categorized from drone video footage. Each tagged whale had a different coloured animal-borne tag that was visible from the drone video to confirm the whale identification matching the drone to the correct tag. We used the drone video footage to visually count the total breaths per surface interval and categorize the behavioural state of each individual dive the whales performed (Fig 1 and Table 2). We defined each dive as being either resting, travelling, or foraging using modified behavioural definitions previously described for resident killer whales [8,9,25]. We excluded socializing and milling from our analysis because they were not mutually exclusive to the other behavioural states, and rarely occurred among the eight tagged animals with drone footage. As well, we observed and recorded when logging behaviour (i.e. when whales remain relatively motionless on the surface) occurred on drone; however, this behaviour rarely occurred and therefore was not included as a behavioural state in further statistical comparisons.

**Table 2:** Defined behavioural states of northern and southern resident killer whales observed on drone video.

Behavioural state	Definition
Travelling	Moving in the same general direction between surfacings at a constant pace of moderate speed, often with other whales present in a group. Very consistent dive and surface interval durations with more consistent depth. Max dive depth travelling was qualitatively less variable compared to foraging.
Foraging	Not moving in the same general direction and not moving at a constant pace of moderate speed; often with longer dives between surfacings; often dolphins are present; often the focal whale is alone or with few other whales. Max dive depth foraging was more variable than travelling based on video observations. Often observed whale’s body arching at the surface before performing a deep dive indicated by losing visibility of whale below surface.
Resting	Swimming at a very slow speed with other whales present in a group; not making any significant progress in one direction; includes shallow diving.
Logging	Stationary at the surface with other whales present in a group; synchronized breathing within the group; does not include shallow diving and surfacing (blowhole may or may not be submerged; a portion of the dorsal fin remains above the water).

Travelling, foraging, resting, and logging were defined according to definitions used in previous killer whale studies [8,9]. See methods for details.

If the behavioural state observed at the surface on the drone video did not meet the defining criteria in Table 2, we classified those surface intervals as “unidentified” and removed them from analysis (n = 28 total surface intervals removed as unidentified). These “unidentified” behavioural states comprised a comparatively small proportion of all behavioural states observed on the drone video (5.6% out of 504 total respirations were unidentified). The unidentified behavioural states appeared to occur randomly among seven of the eight animals with available drone video.

#### Time-depth and video data processing and synchronization

Dives and surface intervals were defined for all dives observed on both drone footage and animal-borne tags. Dives and surface intervals were defined with a 0.5 m minimum dive threshold on time-depth data at 2 Hz [26]. The dives and surface intervals identified in the drone videos were matched to corresponding dives on the animal-borne tags in R based on the start and end times of the individual dives. Accuracy of matching was verified by visual plots for each animal for all dives. Additional details about the drone and TDR data processing and synchronization are contained in S1 Appendix.

### Statistical analysis

#### Hidden Markov models to predict track behavioural states

Drone video allowed us to visually determine the behavioural state associated with many dives, but we only had drone video observations on ∼ 6% of the total dives that killer whales performed (n dives with video = 476; n total dives on 11 whales = 8118, Table 1). We therefore developed and fitted hierarchical hidden Markov models (HHMMs) that used animal-borne time-depth data along with behavioural states directly observed on the drone video to predict the behavioural states of a dive track in a single hierarchical process [14]. We defined a track as the shortest continuous sequence of complete dives and surface intervals that was **≥** 10 min in cumulative duration (i.e., the sum of all the individual dives and surface intervals in that track). The HHMM modeled the sequence of track behaviours as a partially observed Markov chain and modeled the sequence of individual dives within a track as another partially observed Markov chain whose dynamics depended upon the behavioural state of that specific track. As such, the sequence of dive types within a track was dictated by the behavioural state of that track as a whole.

We set 10 minutes as the track threshold duration to allow sufficient time for a killer whale to balance its O_2_ stores [17–19], and ensure that we did not overestimate respiration rates [3,4,8]. This extended time limit incapsulated the potential for whales to carry an oxygen debt over multiple dives that is not fully repaid during a single surface interval. Subsequent analysis only used data calculated over **≥** 10-min tracks to focus on meaningful respiration rates calculated over **≥** 10-min tracks.

A detailed description of the HHMM model and dive characteristics of individual dives from drone video are described in S2 Appendix. In brief, we sought to predict three track-level behavioural states (resting, travelling, and foraging) by first using behavioural state labels identified through drone video on a subset of tracks (i.e., these were observed labels from drone video, not unobserved or predicted from the HHMM). We then summarized each individual dive into a dive type using its maximum dive depth (m), dive duration (s), and surface interval duration (s) as calculated from the animal-borne tags. These dive types were identified as shallow, medium, and deep. Within the HHMM, we used these TDR parameters combined with known behavioural states observed on drone video to predict the behavioural states of all dive tracks (Table A in S2 Appendix). The individual logging occurrences we observed and recorded with the drone were also incorporated into the HHMM model as dive types (similar to our shallow, medium, and deep dive types); however, the tracks as a whole were labelled as either resting, travelling, or foraging based on the individual dive types and observed behavioural labels within that track.

**Fig 2:**
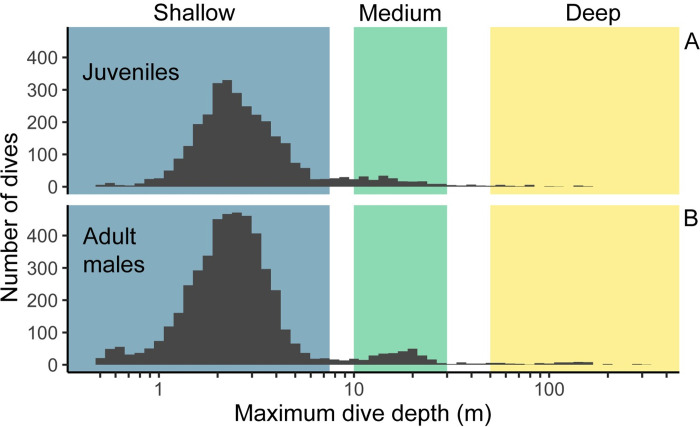
Histogram of maximum dive depths for HHMM dive types. Note that dive depths are plotted on a log scale for 4 adult male (n = 4955 dives) and 7 juvenile resident killer whales (n = 3163 dives).

Resident killer whales primarily hunt for salmon at deep depths [10]. We therefore created individual dive-level labels to help the HHMM identify foraging behaviour by visually inspecting the dive profiles and histograms. They revealed three distinct dive types that included dives less than 7.5 m (shallow), dives between 10–30 m (medium), and dives deeper than 50m (deep) (Fig 2). As such, we labeled all dives within these thresholds accordingly. If the maximum depth of a dive occurred between explicitly defined depth categories, we left that dive unlabelled prior to fitting the HHMM. We labelled any track with at least one “deep” dive (> 50 m) as foraging because previous studies have shown that deep dives are more often linked with foraging [10]. Any dive that was deeper than 30 meters and also within 2 minutes of a previously labelled “deep” dive was also labelled as foraging. This is consistent with dive depths associated with foraging [10], and meant that we relied on our HHMM to identify deeper foraging dives when our drone video labels were insufficient. In addition, we labelled dives with surface intervals > 10 s as logging because all video-labeled logging occurrences satisfied this threshold, and all video-labeled non-logging occurrences did not. We incorporated all known labels into the HHMM in a similar manner to Li et al. [27].

We fit separate HHMMs to the adult male and juvenile killer whale data sets. All HHMM analyses were done using the *momentuHHMM* package in R [28]. Dive characteristics input to the HHMM were maximum dive depth, dive duration, and surface interval duration. Since dive labels are directly related to dive characteristics, testing for a difference in the means of any dive characteristics among behavioural states predicted by the HHMM would result in highly inflated type I error rate [29]. Consequently, we did not analyze dive characteristics per behavioural state for tracks predicted by the HHMM, and subsequent plots of individual dive durations were exploratory only.

#### Error in HHMM predicted behavioural states

We assessed the error of the HHMM in predicting track behaviour using k-fold cross-validation, where one “fold” in the cross-validation scheme corresponded to a single whale’s dive profile with k = 11 total whales [30]. For each whale, a new HHMM was trained using all of the data except for that whale, and the left-out whale’s behaviour was predicted using the Viterbi algorithm [31] with the newly-trained HHMM. The true track labels from drone video were then compared with the predicted track labels generated from the Viterbi algorithm. This procedure predicted the model’s ability to accurately label the behaviour of a new whale if that whale had no video-generated labels. This procedure yielded one confusion matrix (contingency matrix) per coarse-scale behavioural category (foraging, resting, and travelling) per sex and age category (there were two males and six juveniles with video data). Each of the six contingency matrices included the total number of video-labelled tracks. That is, error was only assessed on tracks that had video labels (juveniles = 66 tracks video-labelled out of 176 total tracks predicted; males = 62 tracks video-labelled out of 243 total tracks predicted).

Each confusion matrix contained counts of four scenarios: true positives (TP), true negatives (TN), false positives (FP), and false negatives (FN) (see Table 3 for definitions of these terms). These counts were used to calculate detection (TP rate), false positive rate (FP rate), precision, specificity (TN rate), and accuracy across behavioural states for both sexes (Table 3). Detection rate is the proportion of times that the HHMM model correctly determined that the behaviour had occurred, out of all times that the behaviour occurred. The detection rate was calculated as TP/(TP+FN). This calculation was repeated separately for each behaviour and sex. For example, the detection rate for foraging in females is the proportion of foraging tracks on drone video that were correctly classified as foraging tracks by the HHMM model. A false positive occurred when the HHMM identified a track as a specific behavioural state, but the drone video observed any behaviour except that target behaviour. It was calculated as FP/(FP+TP). Precision was the proportion of times that the HHMM correctly determined that the behaviour had occurred, out of all times that the HHMM determined that the behaviour occurred and was calculated as TP/(TP+FP). Specificity, the proportion of times that the HHMM correctly determined that the behaviour had not occurred, out of all times the behaviour had not occurred, was calculated as TN/(TN+FP). Accuracy, the proportion of the total tracks that the HHMM correctly predicted the behaviour, was calculated as (TP+TN)/(TP+TN+FP+FN).

**Table 3:** Contingency matrix used to assess how well the HHMM predicted the behavioural state of killer whales observed on drone video.

	Drone video observed (Truth)	HHMM predicted (Estimate)	Description
**True Positive (TP)**	Yes	Yes	Both drone video and HHMM identified behaviour of track as foraging.
**True Negative (TN)**	No	No	Both drone video and HHMM identified behaviour of track as any behaviour *except* foraging.
**False Positive (FP)**	No	yes	HHMM identified track as foraging, but drone video observed any behaviour *except* foraging.
**False Negative (FN)**	Yes	No	HHMM identified behaviour of track as any behaviour *except* foraging, but drone video observed foraging

Drone video observations of behavioural states (“Truth”) were compared to HHMM predicted behavioural states (“Estimate”) using a k-fold cross-validation on animals that had concurrent video to yield a confusion matrix (contingency table) per behavioural state for foraging, resting, and travelling tracks. This contingency table example was used to measure errors in identifying foraging behaviour and was repeated for resting and travelling. Sample size of whales for all behavioural states was n = 7 juveniles and n = 4 adult males.

#### Respiration rates

We used a track of dives as the unit of analysis to calculate the average respiration rate over a physiologically meaningful time span [≥ 10 min; 3,4,8] while also incorporating variation in diving behavioural states observed in the field. Respiration rate (breaths min^-1^) was calculated as the total number of respirations (e.g., total number of surface intervals as we confirmed 1 breath = 1 surface interval) per track divided by the cumulative track duration on the animal-borne tag. The respiration rates were then compared between track behavioral states within each sex.

All respiration rate analysis was done using the track behavioural states predicted by the HHMM for all of the dives recorded by the animal-borne tags. We performed a repeated measure ANOVA using linear mixed-effects models [nlme package, LME; 32] to determine if the average respiration rate (breath min^-1^) within a track varied among behavioural states predicted from the HHMM model (resting, foraging, or travelling). Since the independent variable is categorical and we have multiple tracks per individual whale, we included Whale ID as a random effect. As such, our modelling took into account the dependence in the data gathered from an individual, and also related each animal to other animals by expressing individual animal variation relative to the mean of the population [33,34].

The statistical significance of behavioural state was determined using a conditional ANOVA F-test. Model comparisons were performed using a likelihood ratio test (LRT) on two hierarchically nested models. When models were significant, Tukey post hoc tests with Bonferroni adjusted p-values were used to compare the means between multiple levels and identify the behavioural state(s) that differed [mvtnorm and multcomp R libraries; 35,36]. Statistical significance was set at α = 0.05. Adult males and juveniles were modelled separately for all HHMM and LME models due to differences in predicted energetics related to body mass.

#### Dive durations

We divided all individual dive durations on CATS tags into short (< 1 min) or long dives (**≥** 1 min) to explore diving patterns based on behavioural states predicted by the HHMM. This distinction was for exploratory purposes to give context to calculated respiration rates, and was based on the statistical distributions of dive durations, and not on any physiological rationale. Our 1-minute breakpoint is the same as used by Baird et al. [37] to differentiate short and long dives of southern resident killer whale carrying time-depth recorders—and is very close to the 57.77-second breakpoint that Miller et al. [9] discovered using a log frequency analysis of all dive durations made by transient killer whales equipped with Dtags. These two analyses of diving behaviours of killer whales are consistent with our treatment of the killer whale dive data, and our definitions of short and long dives.

All dive durations were measured directly by time-depth recorders (CATS) and were grouped according to the behavioural states predicted by the HHMM (see S2 Appendix for details). We calculated mean dive durations of short and long dives, as well as the duration of all dives by behavioural states for males and juveniles (see S1 Table for values). Note that the distinction between short and long dives was not included in the HHMM or respiration rate analysis. Note also that respiration rate analysis was done on a different level of the dataset (i.e., only on tracks of dives > 10 min, and not on individual dives).

#### Estimated oxygen consumption rate

We calculated the rate of oxygen consumption (VO_2_, L O_2_ min ^-1^) for use in bioenergetic models and comparison with published values [2,7]. This was done using physiological data from trained killer whales [3], along with predicted body masses (see S3 Appendix) and mass-specific tidal volumes (V_T_). In brief, we determined body lengths for each of the animals we tagged in 2020 using a Gompertz growth model per sex [38]. We then estimated body mass (kg) as a function of body length (cm) for each individual whale [39]—and matched the tagged whales in our study to whales in Kriete (Table 16 in Kriete [3]) that were within 15% similar body mass and also the same age class per sex to yield predicted mass-specific V_T_ per whale per behavioural state (Table 1 in Kriete [3]). Mass-specific V_T_ also yielded estimates of mass-specific oxygen extraction from inhaled air (E_O2_) and the concentration of oxygen dissolved in blood and tissue (T_O2_) as well. Resting E_O2_ came from trained killer whales while they rested (activity level 1)—while foraging and travelling E_O2_ were estimated from trained animals undertaking light to moderate swimming and shallow diving activities (activity level 2, Table 9 in Kriete [3]). Animals A113, I129, I145, I107, and D21 were excluded from VO_2_ calculations because their predicted body masses were not within 15% of the predicted body masses and V_T_ of the killer whales in Kriete [3]. Additional details regarding calculations, as well as predicted body length (cm), predicted body mass (kg), and mass-specific tidal volume (V_T_) per whale used to calculate VO_2_ are contained in S3 Appendix.

## Results

### Summary of data collected

Drone video data subsampled 6% of the 8,118 total dives with available time-depth data across all 11 killer whales (Table 1). For juveniles, the majority of behavioural states observed on the drone video at the level of individual dives were travelling (78.1%), followed by resting (12.9%), foraging (7.2%), and logging (1.8%, n = 389 total dives). In contrast, foraging was observed more often on drone video for adult males (58.6%) compared to the other behavioural states (rest = 37.9%, travel = 2.3%, logging = 1.2%, n = 87 dives).

Activity budgets from drone video are a discontinuous subsample of the total behaviours on animal-borne tags. For juveniles, we used 389 behavioural labels from the drone videos to inform the HHMM model, which yielded 191 total tracks prior to filtering for only tracks that were ≥ 10 min. For the adult males, 87 behavioural labels from drone video were used to inform the HHMM model, which yielded 279 total tracks prior to 10-minute track duration filtering.

### HHMM predicted track behavioural states

**Fig 3:**
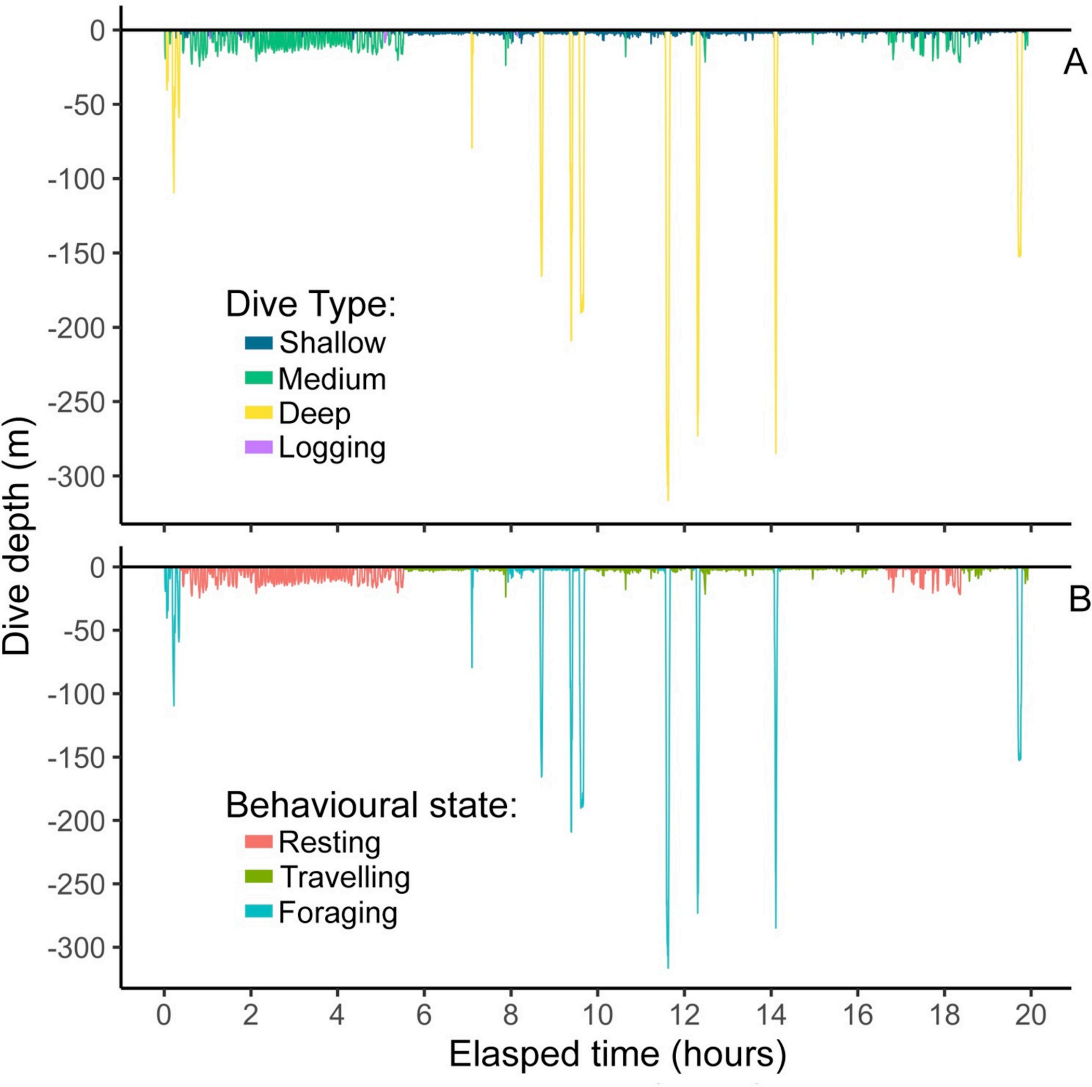
Example of a dive-depth profile from adult male whale D21 illustrating depth categories and track behavioural states predicted by the HHMM. Panel A represents the depth categories used to classify behavioural states within the HHMM. On D21, sample sizes were logging (n = 27), shallow (< 7.5m, n = 1,687) medium (10–30 m, n = 87), and deep (> 50m, n = 11, see S2 Appendix for details). Panel B represents predicted behavioural states by the HHMM for resting (n = 550 dives), travelling (n = 1005 dives), and foraging (n = 257 dives) on this animal. This adult male whale made 1812 total individual dives recorded on the animal-borne tag.

For juveniles, the HHMM identified 191 tracks from 3,153 individual dives (n = 7 whales, Table 4 and Fig 3) of which there were fewer occurrences of foraging (n = 40 tracks) compared to travelling (n = 75) and resting (n = 76). In contrast, the male HHMM classified 4,955 individual dives into 279 behavioural-specific tracks (n = 4 whales, Table 4 and Fig 3) of which occurrences of resting (n = 100 tracks) and travelling (n = 103) were also similar to one another, but occurred more frequently than foraging (n = 76). Excluding tracks < 10 minutes in cumulative duration reduced track sample sizes by 8–13% but did not considerably affect the relative numbers of resting, travelling, and foraging tracks available for further analysis of male and juvenile respiration rates. Activity budgets at the level of the tracks showed both groups of killer whales spent similar proportions of time resting (juveniles = 39.8%, males = 37.0%) and travelling (juveniles = 38.6%, males = 37.5%), and less time foraging (juveniles = 21.6%, males = 25.5%, Table 4).

**Table 4:** Total number of tracks and activity budget per behavioural state of resident killer whales predicted by the hierarchical hidden Markov models (HHMM).

	Behavioural state	Predicted by HHMM (counts)		Activity budget (%)	Cumulative duration of tracks (≥ 10 min)		
		Total	≥ 10 min	Mean	Mean	S.D.	Range
**Juvenile**	Resting	76	70	39.8	11.6	1.5	10.0–16.0
	Foraging	40	38	21.6	11.2	1.6	10.0–17.2
	Travelling	75	68	38.6	10.6	1.1	10.0–15.0
**Adult Male**	Resting	100	90	37.0	10.9	1.0	10.0–13.9
	Foraging	76	62	25.5	11.1	1.7	10.0–16.9
	Travelling	103	91	37.5	10.3	0.3	10.0–11.3

Only tracks that were **≥** 10.0 minutes were used in the subsequent respiration rate analysis (juveniles = 176, and adult males = 243 tracks). Sample size of whales for all behaviours was n = 7 juveniles and n = 4 adult males.

### Error in HHMM predicted behavioural states

A k-fold cross-validation that compared the “true” behavioural state observed on drone video to the “estimated” behaviour predicted by the HHMM indicated that the model reliably predicted when a killer whale was foraging, resting, or travelling at the level of the track with an accuracy of 85% for juveniles and 96–98% for males (Table 5). However, for the juveniles, detection rate (true positive rate) was highest for foraging (95%), followed by travelling (71%), and then resting (56%). For males, detection rate was slightly higher for resting (100%) compared to foraging (96%), but this was influenced by low or absent numbers of false positives and false negatives across all behaviours. There were no true positive predictions for males travelling, and consequently no detection rate could be calculated for travelling males. It is important to note that although we had more juvenile killer whales with video than males (6 vs 2 whales, Table 1), we had more individual dives (3,163 vs. 4,955) and more total tracks for males than juveniles (243 vs 176, Table 3).

**Table 5:** Summary of HHMM measures of error predicting behavioural state of killer whales relative to drone video observations.

		Total count				Measures of error (%)				
		TP^*^	FN^*^	FP^*^	TN^*^	Detection	FP Rate	Precision	Specificity	Accuracy
**Juvenile**	Resting	5	4	6	51	56	55	45	89	85
	Foraging	21	1	9	35	95	30	70	80	85
	Travelling	25	10	0	31	71	0	100	100	85
**Adult** **Male**	Resting	5	0	1	56	100	17	83	98	98
	Foraging	55	2	0	5	96	0	100	100	97
	Travelling	0	0	1	61	NA	100	0	98	98

True behavioural state labels from drone videos were compared with predicted labels generated from the HHMM to assess the model’s ability to accurately label the behaviour of a new whale without video-generated labels. Measures of error were calculated at the level of the track as percentages and included detection (TP/(TP+FN)), false positive rate (FP/(FP+TP)), precision (TP/(TP+FP)) specificity (TN/(TN+FP)) and accuracy ((TP+TN)/(TP+TN+FP+FN)). Error was only assessed on tracks that had video labels (juveniles = 66 video-labelled out of 176 total tracks predicted; adult males = 62 video-labelled out of 243 total tracks predicted). Sample size of whales for all behaviours was n = 7 juveniles and n = 4 adult males.

^*^ TP = true positive, FN = false negative, FP = false positive, TN = true negative. See Table 3.

### Respiration rates

**Fig 4:**
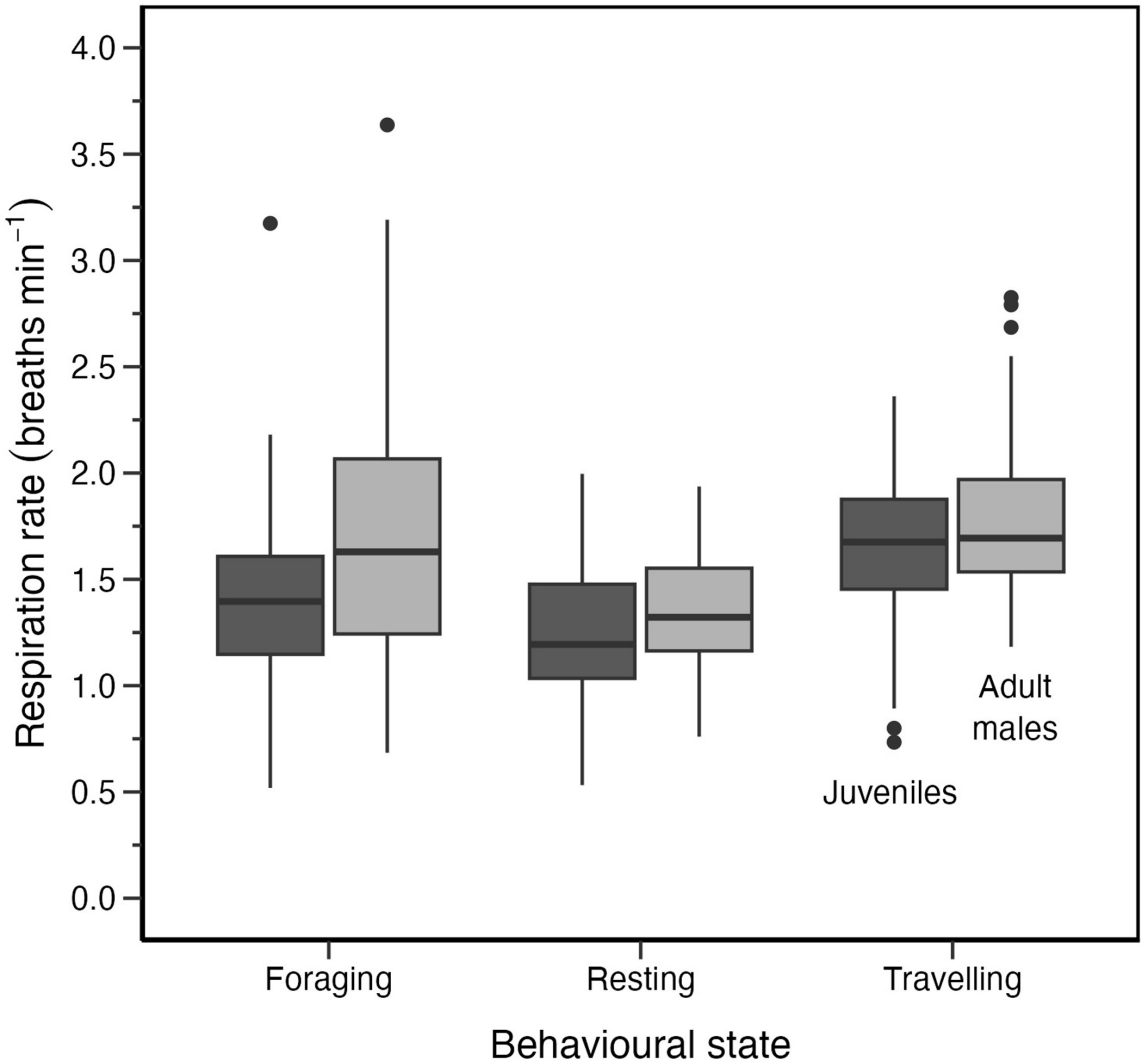
Respiration rates (breaths min^-1^) of 7 juvenile and 4 male resident killer whales while resting, foraging, and travelling. The number of tracks were resting (70), foraging (38), and travelling (68) for juveniles compared to resting (90), foraging (62), and travelling (68) for adult males. Sample size of whales across all behaviours was n = 7 juveniles and n = 4 adult males.

The killer whales observed on the drone video breathed once per surface interval for all behavioural states of interest (resting, travelling, and foraging). The only exception was logging where the whales took more than one breath per surfacing (mean logging = 2.1, range = 2.0–3.0 breaths). Respiration rate (i.e., the number of surface intervals divided by track duration) varied significantly among track behaviours for juveniles (Fig 4, LRT = 11.28, p = 0.004) and males (Fig 4, LRT = 64.03, p < 0.001). Mean respiration rate for juveniles was highest during travelling tracks (1.6) compared to foraging (1.5) and resting (1.2 breaths min^-1^). Males showed similar patterns with the highest mean respiration rate occurring during travelling (1.8) followed by foraging (1.7) and resting (1.3 breaths min^-1^).

Post-hoc Tukey tests, indicated that average respiration rates of travelling tracks differed from those of resting tracks for juveniles (Tukey, p = 0.003), while for males such differences were across travelling and both foraging (Tukey, p < 0.001) and resting (Tukey, p < 0.001). All other comparisons in the Tukey tests showed no significant differences between juveniles and males. We did not make any further comparisons between males and juveniles results because we used a separate HHMM to predict their track behavioural states.

### Dive durations

**Fig 5:**
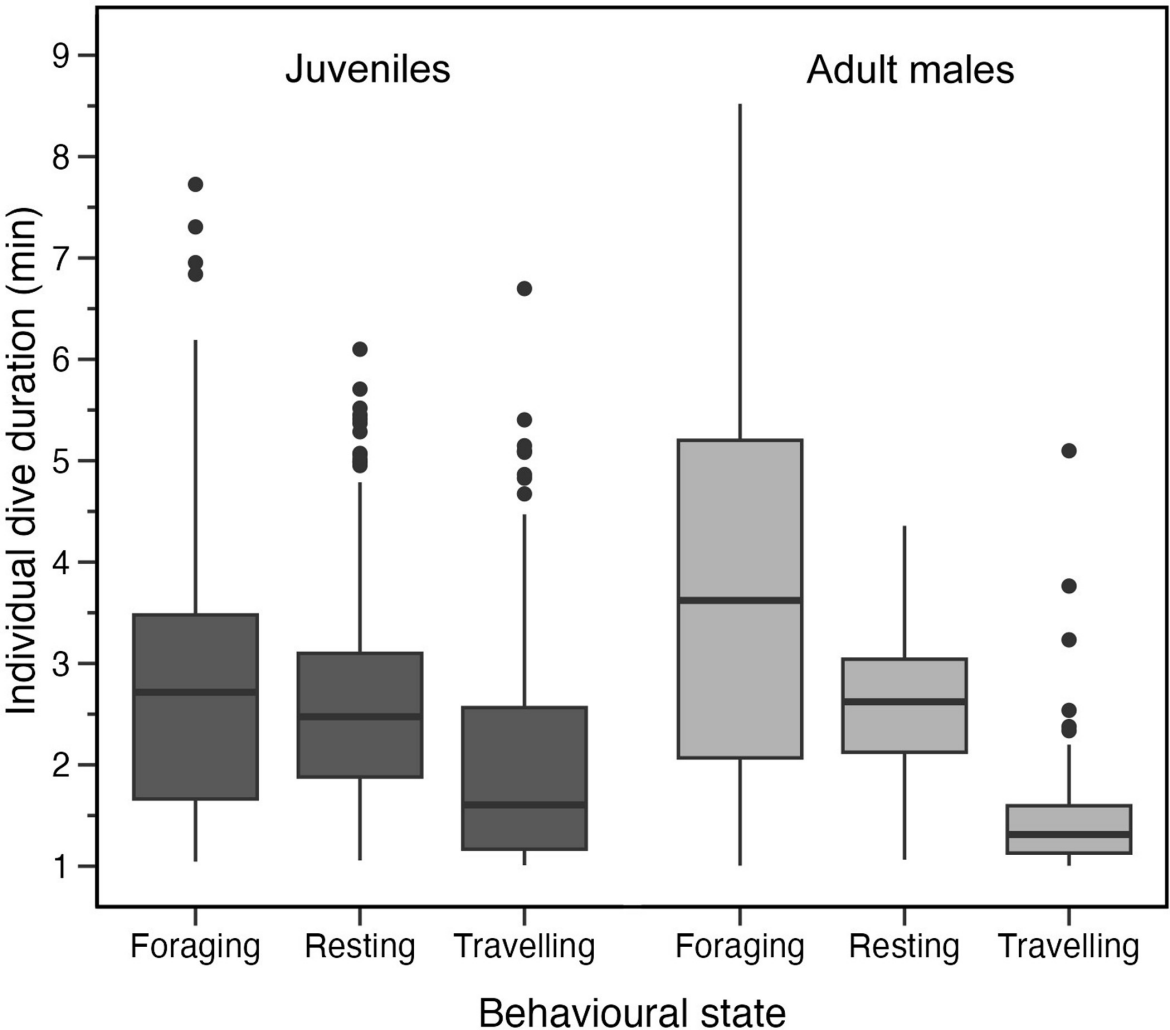
Individual long dive durations (≥ 1 minute) of 7 juvenile and 4 adult male resident killer whales for different behavioural states (foraging, resting, and travelling). Individual dive duration was recorded by the CATS tags with behavioural state as predicted by the HHMM. Long dives include 11% of the total dives in juveniles (n = 354 long dives) and 9% of the total dives in adult males (n = 440 long dives). Sample sizes for total individual dives ≥ 1 minute were n = 76-foraging, 190-resting, and 88-travelling dives for juveniles; and n = 93-foraging, 241-resting, 106-travelling dives for adult males.

**Fig 6:**
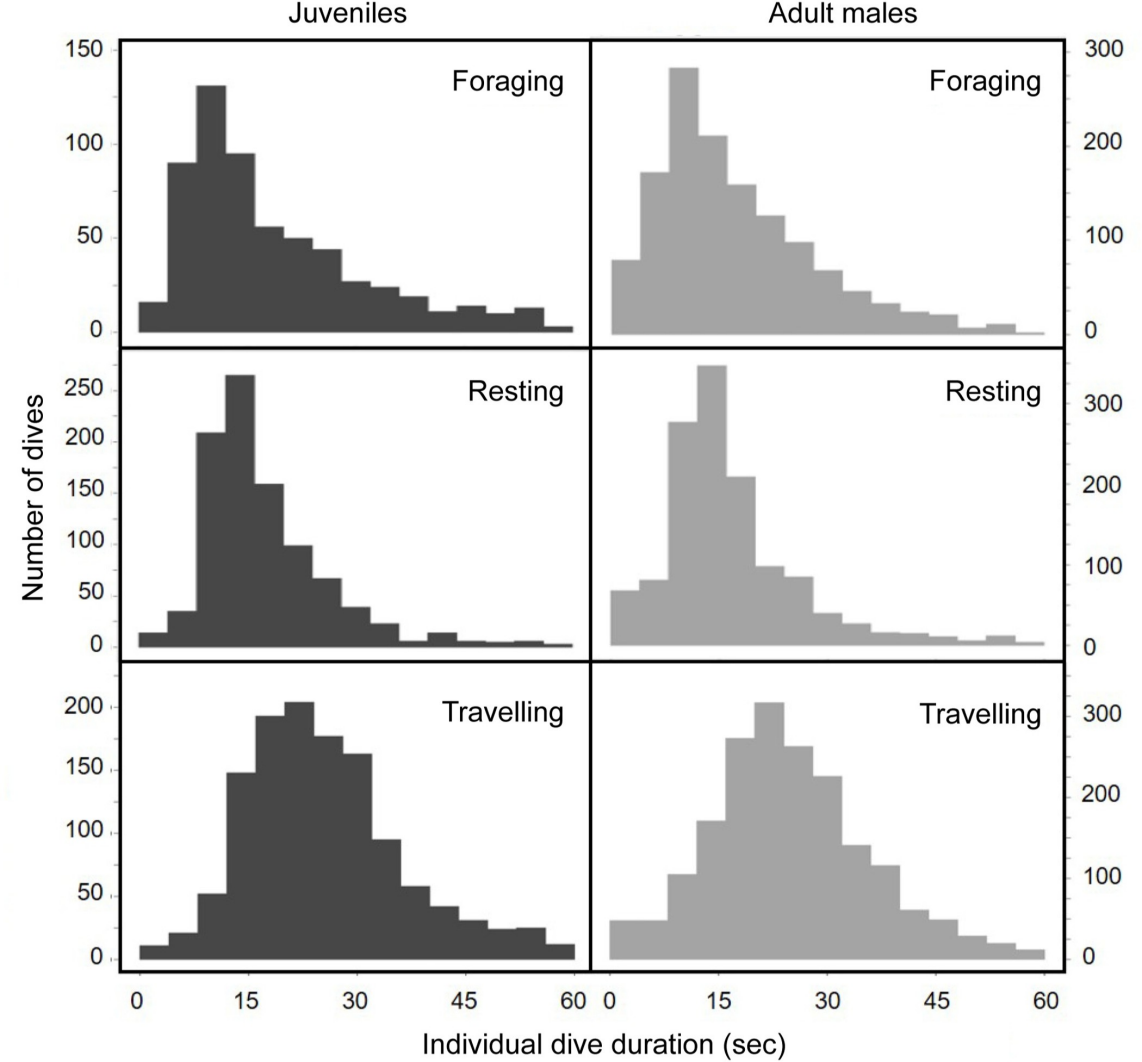
Distributions of individual dive durations for short dives (< 1 minute) from CATS tags with behavioural state predicted by HHMM (bins = 4 seconds). Individual dive duration was recorded by the CATS tags with behavioural state predicted by the HHMM. Short dives were 89% of the total dives in juveniles (n = 2899 short dives) and 91% of the total dives in adult males (n = 4515 short dives). Sample sizes for total individual short dives < 1 minute are n = 603-foraging, 950-resting, and 1256-travelling dives for juveniles; and n = 1340-foraging, 1296-resting, 1879-travelling dives for adult males.

Individual dive durations for both adult male and juvenile whales ranged from a few seconds to as long as 8.5 minutes for the males, and 7.7 minutes for the juveniles (all dives recorded by the animal-borne tags as shown in Figs 5 and 6)—and averaged 0.5 min (32 sec, SD = 0.78, n = 4,955 individual dives) for males and 0.6 min (36 sec, SD = 0.84, n = 3,163 individual dives) for juveniles. The majority of all individual dives on animal-borne tags were < 1 min for both juveniles and males (89% of juvenile dives, and 91% of all individual male dives on animal-borne tags).

In terms of dives **≥** 1 minute (Fig 5 and S1 Table), the adult males we tagged made longer foraging dives on average (mean = 3.8 min for males vs 2.9 min for juveniles), and had shorter travelling dives than did the juveniles (mean = 1.5 vs 2.1 min), but had similar individual resting dives (mean = 2.6 vs 2.7 min). Long dives (i.e., **≥** 1 minute) accounted for 11% of the total dives made by juveniles (n = 354 long dives) and 9% of the total dives made by males (n = 440 long dives). Individual dive durations for adult males were also more variable compared to those of juveniles for all behaviours (i.e., while foraging, resting or travelling; Fig 5). In terms of long dives (i.e., **≥**1 min), juvenile dive durations appeared to conform more to a central tendency than did those of males for all behavioural states. However, adult males and juveniles were consistent in terms of making longer foraging dives on average than resting dives, which were in turn longer on average than dives made when travelling.

Shorter duration dives (< 1 min) tended to look more normally distributed while travelling, but were skewed while foraging (Fig 6). The distribution of short resting dives made by adult males and juveniles fell between those they made while foraging and travelling. Overall, however, the distribution of short-duration dives (< 1 min) for all three behavioural states were consistent between adult males and juveniles (Fig 6), unlike their longer duration dives (**≥**1 min) that showed greater variability among behavioural states and among males and juveniles (Fig 5). The average durations of short dives were similar between adult males and juveniles, but became increasingly shorter as the behaviours of the whales shifted from travelling (26 and 25 sec for males and juveniles, respectively), to foraging (19 vs 17 sec), and resting (17 vs 17 sec) (S1 Table).

In terms of all dives combined (i.e., short + long dives), average dive times of adult males were 28 sec while travelling, 31 sec while foraging, and 38 sec while resting (S1 Table)—all of which were longer for each behavioural state than for juveniles, which averaged 32 sec while travelling, 36 sec while foraging, and 31 sec while resting. In terms of the distribution of dive types by behavioural categories, the greatest proportion of long dives occurred while the killer whales travelled (15.7% for adult males, and 16.7% for juveniles), and the smallest proportion of long dives occurred while they foraged (5.3% for males vs 6.5% for juveniles). The most notable difference between adult male and juvenile dive durations were associated with resting. Males made proportionally fewer long dives while resting than did juveniles (6.5% vs 11.2%).

### Estimated oxygen consumption rate

We calculated VO_2_ only for animals that had predicted body masses within 15% of the body masses and matched age classes available in Kriete [3, see S3 Appendix for details]. Calculated VO_2_ rates ranged from 2.5–18.3 L O_2_ min^-1^ for juveniles and 14.3–59.8 for adult males across all behavioural states (Table 6). Mean calculated VO_2_ for juveniles was highest while travelling (7.4), followed by foraging (7.3), and was lowest for resting (6.7 L O_2_ min^-1^_,_ Table 6, LRT = 13.26, p = 0.0013). Post hoc Tukey tests showed that only foraging VO_2_ differed from resting (p < 0.001). All other comparisons in the Tukey tests within juveniles were not significant indicating that the most prominent difference was only between resting and foraging. For adult males, VO_2_ significantly varied among behavioural states (LRT = 110.49, p < 0.001) and was significantly higher for travelling (43.6) followed by foraging (35.2), then resting (25.8 L O_2_ min^-1^_,_ Table 6, Tukey, p < 0.001 for all comparisons).

**Table 6:** Example of calculated oxygen consumption rates (VO_2_) and respiration rates of 6 resident killer whales derived from behavioural data at the level of the track with mass-specific tidal volumes per sex and age class.

	Behavioural state	Tracks	Calculated VO_2_ (L O_2_ min ^-1^)^b^			Respiration rates used in VO_2_ calculations (breaths min^-1^)^a^		
		N	Mean	S.D.	Range	Mean	S.D.	Range
**Juvenile**	Resting	38	6.7	4.0	2.5–18.3	1.2	0.3	0.7–1.8
	Foraging	14	7.3	1.9	3.9–12.1	1.5	0.3	0.9–2.0
	Travelling	28	7.4	1.8	3.7–11.9	1.4	0.3	0.7–1.9
**Adult Male**	Resting	47	25.8	5.1	14.3–35.0	1.4	0.3	0.8–1.9
	Foraging^c^	7	35.2	9.1	19.3–44.1	1.6	0.4	0.9–2.0
	Travelling	35	43.6	5.8	32.0–59.8	2.0	0.3	1.5–2.7

Sample size of whales for all behaviours was n = 4 juveniles and n = 2 adult males^a^. See Table in S3 Appendix for details on calculations as well as predicted body length (cm), predicted body mass (kg), and mass-specific tidal volume (V_T_) per whale used to calculate VO_2_.

^a^ These respiration rates are different from Fig 4 and the LME analysis because they only include a subset of animals that met VO_2_ criteria. Some animals (A113, I129, I145, I107, and D21) were excluded from VO_2_ calculations and this subset of respiration rate estimates because their predicted body masses were not within 15% of the predicted body masses and sex-specific V_T_ of the killer whales in Kriete [3].

^b^ Oxygen consumption (VO_2_, L O_2_ min^-1^) was calculated per track (only tracks ≥ 10 min cumulative duration) using Equation 1 in Roos et al. [2] with mass-specific values originally measured by Kriete [3]. Maximum tidal lung volume varied by predicted body mass and activity level (Table 1 in Kriete [3]). Mean oxygen extraction from inhaled air (E_O2_) for resting was from activity level 1, and foraging and travelling was from activity level 2 per sex per animal of similar body mass (Table 9 in Kriete [3]). For adult males only, V_T_ and E_O2_ for activity level 2 was averaged from activity levels 1 and 3 values for the killer whale named Hyak because it was not directly measured in Kriete [3].

^c^ VO_2_ for foraging males only includes whale L87 because L88 had no foraging tracks.

## Discussion

Using drones and simultaneously deployed biologging devices to quantify respiration rates and associated behavioural states confirmed that killer whales take a single breath per surfacing while travelling, foraging and resting. Thus, energy expenditure can be indirectly estimated from an assumed amount of oxygen exchange per breath—and from using the numbers of surface intervals recorded by time-depth recorders as a proxy for numbers of breaths taken. In addition, we found that the mean respiration rates estimated from dive data per track for juveniles and adult males while resting, foraging, and travelling were consistent with those reported for other killer whales. We also found that HHMMs can accurately predict behavioural states of unknown dive types but needed to be informed with video verified behaviours to yield biologically meaningful categories. Lastly, we found that VO_2_ predicted from respiration rate significantly varied among behaviours for both adult males and juveniles with both age categories of killer whales spending significantly less energy while resting, and juveniles expending less energy overall than the larger males.

### HHMM predicted track behavioural states

Hidden Markov models have previously been used to predict and categorize movement patterns of several cetacean species. Hidden Markov models have been used to study the movement patterns of blue whales [*Balaenoptera musculus*; 16], long-finned pilot whales [*Globicephala melas*; 13,40], harbour porpoises [*Phocoena phocoena*; 14], sperm whales [*Physeter macrocephalus*; 40], humpback whales [*Megaptera novaeangliae*; 40], northern bottlenose whales [*Hyperoodon ampullatus*; 40], and killer whales [12,15,20,41]. These studies demonstrate that HMMs are a robust method to categorize behaviours using movement data in cetaceans. However, few studies have used independent data to validate the behavioural states predicted by the HMM. We built on the work of these studies by validating all behavioural states from our HHMMs using drone observations and performing a full cross-validation analysis.

Using HHMMs allowed us to increase our statistical power by expanding the number of total dives analyzed while also defining longer duration tracks that included multiple dives to calculate respiration rates. Using drone videos, we only had 476 discontinuous individual dives with observed behavioural states. However, by using drone video to inform the HHMMs, we predicted behavioural states and activity-specific energetics on over 8,000 individual dives with high accuracy (Table 5). Our HHMMs also allowed us to group the 8,000 dives into tracks of at least 10 min to calculate respiration rates over physiologically relevant durations. Thus, using HHMMs as a statistical tool coupled with a subset of drone videos allowed us to predict continuous behavioural states of thousands of previously unknown dives on a free-ranging cetacean.

Our cross-validation comparing the HHMM predictions to the “truth” on drone video indicated that the HHMM had high accuracy, low false positive rates for most behaviours, and high measures of detection indicating that this method is a reliable tool to predict behavioural states and respirations from dive-depth data. Our analysis assumed that all of the predicted behavioural states per track were 100% accurate prior to calculating respiration rate per track. This was a reasonable assumption based on the calculated measures of error (Table 5). Detection was relatively high (71–100%) except in resting juveniles (56%). The lower detection rate for resting juveniles reflects the low total numbers of true positive and false negatives. The HHMM was more reliable at detecting foraging compared to travelling in juvenile whales. This makes sense because we prioritized building the HHMM to detect foraging when deep foraging dives could not be identified from drone video alone. Overall, the false positive rate was low except for juveniles that were resting (55%) and males that were travelling (100%). For travelling males, the high false positive rate was skewed by the absence of true positives and false negatives which prevented calculating detection. Notably, all of the measures of error were influenced by the total number of tracks per behaviour and how many of those individual dives were labelled on drone video per behaviour. In the future, the HHMM could be optimized to maximize or minimize whichever measure or error is of most interest.

### Respiration rates

The mean respiration rates we recorded for juveniles (1.2–1.6 breaths min^-1^) and adult males (1.3–1.8 breaths min^-1^) are similar to those reported over similar durations for other adult killer whales (mean respiration rates of 1.6–1.7 breaths min^-1^ while foraging and travelling, 1.6 breaths min^-1^ while foraging, 1.4 travelling, and 1.0 resting) [4,8]. However, the whales we studied incurred the highest respiration rates while travelling (1.6 juveniles; 1.8 breaths min^-1^ males), followed by foraging (1.5 juveniles; 1.7 breaths min^-1^ males), and then resting (1.2 juveniles; 1.3 breath min^-1^ males).

We had expected foraging costs to have a higher mean respiration rate compared to travelling because foraging presumably requires diving to greater depths for longer periods of time, more non-uniform dive paths, higher levels of body rotations, and greater swimming speeds to capture prey [10]. We also expected oxygen uptake per breath to be greater during foraging relative to other behaviours because each breath taken while foraging reduces the time spent foraging and should therefore carry a high cost compared to breathing while resting and moving slowly near the surface. However, counter to expectations, we found travelling was more energetically expensive than foraging due perhaps to the increased drag and buoyancy costs associated with making shallow dives. Killer whales may also breath less frequently while foraging to optimize their time at depth to encounter and capture prey, and therefore incur an oxygen debt that is not ultimately fully paid back until they travel and rest [17,42–45]. Although we used tracks of ≥10 minutes to allow for oxygen and carbon dioxide balancing, it may not have been sufficient to remove a potential oxygen debt in all tracks. Thus, the 10-minute tracks we used may not have been long enough to include all recovery breaths after deeper foraging dives (see S2 Appendix for more details), and should therefore be considered a minimum foraging respiration rate.

Overall, the adult males had higher mean respiration rates in all behavioural states compared to juveniles as expected because of differences in oxygen storage capacity (lung size) and oxygen use related to differences in body size. However, some of the differences in respiration rates between juveniles and adult males are also likely attributable to behavioural differences between the two age classes as previously discussed.

We were able to incorporate aerial observations of behaviour and breathing with detailed TDR data to yield a more complete picture of whale underwater behaviours. Previous studies calculating respiration rates of killer whales have primarily used surface-based observations from either boats or land to identify diving behaviours and count breaths [4,8]. However, the drone video with TDR data allowed us to directly observe and assign behaviour with greater confidence. It also allowed us to view the same behavioural clip multiple times and at slower speeds. Combining information from drone videos with animal-borne tag data allowed us to build and verify HHMMs to predict behaviours and therefore calculate respiration rates on a large sample of dives. The similarities in the range of respiration rates that we and others have obtained highlights the viability of using HHMMs to calculate respiration rates and determine behavioural states.

### Dive durations

Dive durations differed according to whether the whales were travelling, resting or foraging (Figs 5 and 6 and S1 Table). However, the different distributions of dive durations between behavioural states make physiological sense and give confidence that the HHMM correctly predicted the three basic behavioural states. For example, the short travelling dives were normally distributed (Fig 6), consistent with animals swimming in a predictable sustainable manner. In contrast, the distribution of short foraging dives was highly skewed (Fig 6), consistent with whales having depleted their O_2_ stores and built up CO_2_ as they extended themselves searching and pursuing prey—and then resurfaced to take short breaths that partially replenished their O_2_ stores and offloaded some CO_2_ before returning quickly to depth to continue foraging (see [44] for discussion of this trade-off pertaining to Steller sea lions).

Our findings (Figs 2 and 5 and 6) are also consistent with those of others showing that killer whales of all ecotypes primarily make short (<1 min), shallow (<10 m) dives—and comparatively fewer long (>1 min), deep (>10 m) dives [8–10,37,46–48]. In our case, short duration dives occurred 10 times more frequently than long dives, and constituted about 90% of all the dives we recorded. Variability in the duration of short dives differed by behavioural state—and was greatest while adult males and juveniles foraged, and least variable while they travelled (i.e., dive times were the most consistent)—with short resting dives falling in between (based on the coefficients of variation for each behavioural states, S1 Table).

The long foraging dives we recorded for males (3.8 min average) were longer than those reported by others (2.8 min mean by Baird et al. [37], and 2.9 min median by Wright et al. [10]), which might reflect differences in the relative availability of prey among studies. These estimates are nevertheless of similar magnitudes, as are the mean durations of short dives made by foraging males (17.4 sec) compared to those reported by others (e.g., 19.8 sec median; Wright et al. [10]), which gives added confidence in our methods and calculations. Separating dives into short and long dives using a 1-minute breakpoint reflects the bimodal nature of killer whale dive patterns, and provides a means to calculate meaningful measures of dive durations that can be used to assess the availability of prey and the impacts of human activities on killer whales. This splitting of dive types is superior to determining average dive times for all dives combined, which were only about 30 sec when pooled due to the non-normality of the complete series of dives, and the overly high proportion of short dives [e.g., 8; S1 Table].

Interestingly, all of the dives made by the 11 animals we studied were shorter than the calculated aerobic dive limit (cADL) for killer whales (all of the dives were < 8.5 minutes). The cADL for killer whales based on scaled measurements from bottlenose dolphins [49] was 10.2 minutes for females and 11.8 minutes for males [9]—while the resting-surface cADL for a trained adult male killer whale based on serial blood lactate measurements was 13.3 minutes [50]. Although we do not know if the killer whales in our study were diving with a pronounced oxygen debt or whether they partially replenished their oxygen stores between dives, delphinids are believed to rarely exceed their aerobic capacity, and are believed to use anaerobic metabolism sparingly to prolong dives if necessary after encountering prey [51]. However, smaller individuals such as juvenile killer whales are likely to be more aerobically challenged than the larger and older individuals—and adult males are likely to have considerably more reserves to pursue challenging prey compared to juveniles [9].

The differences in individual dive durations between adult males and juveniles while foraging and travelling likely reflect differences in age class, social relationships, sample sizes per sex, and oxygen storage capacities related to body size. The majority of the juveniles in our study (86%) were skewed towards young individuals < 13 years old (oldest female was 14), but all of the adult males were > 13 years (15–28 years, Table 1)—with predicted body masses ranging from 1,252–2,555 kg for juveniles and 3,382–4,172 kg for males (Table A in S3 Appendix). The greater variability in durations of the longer dives of juveniles compared to adult males may also reflect behavioral differences associated with social responsibilities and age-based relationships of adult male and juvenile resident killer whales (i.e., prey sharing). Some of the differences between adult males and juveniles, as well as among individuals (particularly during travelling) could also reflect differences among pods (Table 1).

### Estimated oxygen consumption rate

Our calculated VO_2_ varied significantly among behavioural states for both juveniles and adult males—and are similar to those previously reported for killer whales of similar ages and sexes. For juveniles, the mean VO_2_ values we calculated across all behavioural states (2.5–18.3 L O_2_ min^-1^, Table 6) were comparable to the ranges reported for 1 trained juvenile killer whale across different activity states (aged 11 years old, approximately 6–48 L O2 min-1, extrapolated from Fig 9 in Kriete [3]). For adult males, our calculated VO_2_ values (15.8–86.1 L O_2_ min^-1^, Table 6) were slightly higher than those observed for trained killer whales across activity states (approximately 6–72 L O2 min-1, extrapolated from Figs 6 and 7 in Kriete [3]). Other studies that have calculated VO_2_ based on speed and respiration rates have found VO_2_ values ranging from 18–30 L O_2_ min^-1^ for a mix of juveniles and adult females, and 30–60 L O_2_ min^-1^ for adult male killer whales (values extrapolated from Figs 3A and 3D for Model 1 in Roos et al. [2] using fixed T_O2_ values from Kriete [3]). Of these calculated VO_2_ values, we suspect those for juveniles are more robust than for adult males because we had more juvenile whales compared to adult males (7 vs 4 whales). However, the sample size of total tracks was greater for males compared with juveniles (243 vs. 176).

The applicability of using mass-specific tidal volumes to calculate VO_2_ hinges on the availability of activity specific V_T_ and E_O2_ from similarly sized animals of the same sex. Currently the only published source of this information in killer whales is Kriete [3], which lacks a wide scope in predicted body masses, especially for male whales. The body masses of juveniles in our study generally matched the predicted body masses of animals with available mass-specific V_T_ (Table A in S3 Appendix). However, only 2 of the 4 adult males in our study were within 15% of the predicted body masses and mass-specific V_T_ available (Table A in S3 Appendix). Using mass-specific V_T_ and E_O2_ would ideally improve the accuracy of these calculations, but data are currently lacking to do this on a wide range of body sizes per sex and age class [3]. This shortcoming highlights the need for more current mass-specific estimates of respiratory and physiological variables on animals with a wider range of body sizes and ages.

### Study limitations

#### Sampling limitations

In terms of population demographics, our sampled animals were skewed towards immature animals. The majority of the animals in our study were immature and of unknown sex (5 out of 11 whales were unknown and < 13 years old, Table 1). For our study, we grouped our one young adult female into our juvenile category as it is not possible to do an HHMM cross-validation or compare respiration rates among females with only1 adult female. While this choice was not ideal, we note that R48 was a very young adult based on female killer whales reaching maturity between 12–17 years of age once they have had their first calf. Based on R48’s age and size, it is reasonable to assume that her behaviour and physiology were similar to the other juveniles in the HHMM as shown by the similar dive profiles of R48 and R58 (see S2 Appendix for additional explanation concerning our sex and age class grouping decisions). We had more juvenile animals than adult males, but more total dives and total tracks for the males than juveniles, credited largely to an overnight deployment on D21 with 1,812 dives and a longer deployment on I107. The LME models on respiration rate accounted for unbalanced samples between males and juveniles and among behavioral states with whale as a random effect.

The primary constraint we faced in sampling deeper dives with drone video was the challenge of the drone pilot to track and view the surfacing of focal animals following longer and deeper dives (because animals making deep dives usually surfaced out of drone view). The maximum dive depths recorded by animal-borne tags for foraging dives that also had accompanying drone video footage (range 0.6–58m, Table A in S2 Appendix) were substantially less than the maximum dive depths records on our animal-borne tags (range = 0.5–317 m). As a consequence, the drone video data collected were likely biased towards sampling shallower dives.

#### HHMM and LME limitations

Our HHMMs make several assumptions that may have been violated in this dataset. First, the HHMMs treated a behavioural track as the shortest sequence of dives that lasted for at least 10 minutes. We selected this minimum track duration because previous research on killer whales has shown that calculating respiration rates on shorter durations is biased towards overestimating breathing rates [3,4,8]. However, the true underlying behaviour of a killer whale may have changed within the pre-defined “track of dives”, which may have resulted in errors in our parameter and behavioural estimates. Future studies may allow the coarse-scale behavioural state to change at more flexible intervals than the strict 10-minute divisions we set here.

The HHMMs also assumed that the behaviours of all whales of the same age class were identical to one another. However, behaviour often varies between individuals of the same age class. Future studies may incorporate random effects into the HHMMs to account for differences in behaviour among individuals [52]. While our HHMMs assumed that a given dive type (shallow, medium, or deep) had the same emission distribution between all behavioural states, the duration of short dives appears to vary among behavioural states (Fig 6). Future studies can explicitly model the difference in the distribution of dive duration among behavioural states.

The LME model and calculation of respiration rates assumes that the HHMM is completely accurate in predicting the track behaviour. Our cross-validation results showed that the HHMM had an accuracy of 85–98%, supporting the use of this simplifying assumption (Table 5). However, these cross-validation results indicate that there is unaccounted uncertainty in behavioural state predictions, and error metrics associated with respiration rate should be interpreted conservatively.

#### Oxygen consumption assumptions and limitations

We made several reasonable assumptions to calculate VO_2_ from respiration rate as has been done in other studies of large cetaceans [4,53–55]. For example, we assumed that every breath had a constant V_T_, E_O2_ and consequently fixed T_O2_ per breath. We also had to make assumptions to predict oxygen consumption from respiration rate that reflect the challenges of measuring physiological variables on free-ranging cetaceans—while recognizing that the physiological assumptions required to calculate VO_2_ from respiration rate vary by age, and activity intensity within each behavioural state [6]. Thus, while we assumed that maximum V_T_ per breath was likely met during higher energy activities, we recognize that it may be more reasonable to assume that V_T_ and E_O2_ are constant for behaviours that are less energetically demanding or exclude deep dives such as logging or resting. We attempted to correct for such considerations by using specific E_O2_ estimates that corresponded to the behavioural state definitions instead of applying the same E_O2_ and T_O2_ to all behaviours. In the future, direct measurements of O_2_ exchange from whales of a wide demographic range engaged in diverse behaviors will be needed to validate our estimates.

### Application of findings

For the purposes of estimating respirations from only surface intervals recorded by TDR data, the assumption that one breath is equal to one surface interval is valid for resting, foraging, and travelling behavioural states. The only behavioural state that did not have one breath per surface interval was logging, which was rare (1.7%) and occurred at a frequency of 2–3 breaths per single surface interval. However, this may not be a realistic estimate given the relatively few intervals of logging we observed, and the likelihood that there may be more variability in the time that killer whales can spend motionless than what we were able to observe. Nevertheless, we can safely conclude that killer whales take more than one breath per surface interval while logging, unlike when they are travelling, foraging, and resting.

The criteria used to define dive phases has a significant influence on estimated surface interval times as well as on the total number of dives, dive duration, and dive depth within a track [56]. It therefore needs to be given careful consideration when interpreting respiration rate and behavioural data. The minimum depth threshold used to define the start and end of a dive is the second step in defining dive phases (after zero-offset correction), but selected values vary widely among studies even for the same species. Studies that focus on surface behaviours and respirations often use a shallower minimum dive threshold than those that focus on dive behaviour at depth [10,40,56].

Our analysis of the drone footage and TDR data indicate that the minimum dive depth threshold for killer whales should be 0.5 m (Fig 2). This is shallower than the deeper minimum dive depth thresholds used in others studies of killer whales of 1.0 m [10], 1.5 m [56], and ∼1–2 m (dive threshold visually inferred from Fig 2 “shallow dives” [2,9]). We chose 0.5 m as the minimum dive threshold because it allowed us to accurately match all of the surface intervals and associated breaths on drone video to the animal-borne tag (tested depths: 0.5–2.0 m). Deeper thresholds excluded some of the surface intervals which would have underestimated respiration rates in our study. We suggest using a shallower minimum dive threshold when estimating respiration rates from TDR data because it captures all respirations for resting, travelling, logging, and foraging.

The HHMM trained in our study can be used to accurately predict behavioural states and total respirations per behaviour of unknown dives (i.e., dives without drone video) from historical or future TDR data. We tested the HHMMs ability to predict behavioural states on unknown dives (including 3 animals that had no drone video available), and the HHMM still had reasonable error metrics indicating the robustness of this statistical tool. The HHMM was trained and validated with video, and can now be applied to time-depth datasets to identify resting, travel, foraging, or logging behaviours on TDR data. Concurrent use of drones, biologging devices, and Hidden Markov models are a useful means to accurately quantify respiration rates, behavioural states, and energetics for killer whales.

## Conclusions

The primary goal of our study was to calculate behavioural-specific respiration rates needed to estimate energy requirements of killer whales as a function of time spent resting, travelling and foraging. Combining video of verified behaviours from the air with the predicted behavioural states derived from HHMM found that that killer whales breathe once per surface interval and that killer whales breathe at faster rates while travelling than when foraging and resting.

While our findings provide a means to derive respiration-based estimates of energy expenditure from biologging data, the HHMM methods we developed and validated have broader implications. Most notably, they revealed that the minimum dive threshold when processing dive data (used to define the start and end of a dive, and define dive phases from time-depth-data) should be 0.5 m, which is much shallower than the 1–2 m threshold depths used in other published studies [10,40,56]. Deeper dive depth thresholds underestimate total surface intervals, total breaths, VO_2_, and ultimately underestimates the predicted energy requirements of killer whales. A second important methodological outcome from our study was the development of a refined and validated HHMM that has a high degree of accuracy in determining what killer whales do based solely on dive-depth dive data (i.e., resting, travelling, and foraging).

Having concurrent drone video and underwater TDR data to assess errors and validate model predictions were key to being able to quantify biologically meaningful behaviors from killer whale dive data. It has yielded a powerful statistical tool that can accurately determine activity budgets from dive-depth data—and provides a means to assess how changes in conditions have affected energetic costs and how killer whales spend their time.

## Supporting information

S1 Table Dive durations summary statistics. (PDF)

S1 Appendix Video and TDR data processing and synchronization. (PDF)

S2 Appendix Hierarchical hidden Markov model details. (PDF)

S3 Appendix Mass-specific oxygen consumption (VO_2 mass-specific_) calculations. (PDF)

S1 Data HHMM behavioural states, HMM output probabilities, and dive characteristics for individual dives and tracks. (CSV)

S2 Data HHMM behavioural states, respiration rate, and dive characteristics per track. (CSV)
