# Correction: Killer whale respiration rates

**DOI:** 10.1371/journal.pone.0327049

**Published:** 2025-06-25

**Authors:** Tess M. McRae, Beth L. Volpov, Evan Sidrow, Sarah M. E. Fortune, Marie Auger-Méthé, Nancy Heckman, Andrew W. Trites

S1 Table and S3 Appendix were uploaded incorrectly. Please see the correct S1 Table and S3 Appendix here.

## Supporting information

S1 Table Dive durations summary statistics. (PDF)

S3 Appendix Mass-specific oxygen consumption (VO2 mass-specific) calculations. (PDF)
