# Supplementary material for: Killer whale respiration rates
Source: PLoS One. 2024 May 15;19(5):e0302758. doi: 10.1371/journal.pone.0302758 (PMC11095751; doi:10.1371/journal.pone.0302758)
Supplement: S1 Table — (PDF) [file pone.0302758.s001.pdf]

S3 Table. Dive duration summary statistics.

Age Class	Dive Category	Behavioural State	Sample Size	Mean dive duration (min)	Mean dive duration (sec)	Standard deviation (sec)	Coefficient of variation (CV)
Juveniles	Short (< 1 min)	Resting	950	0.29	17.4	9.0	52%
		Foraging	603	0.31	18.6	12.6	68%
		Travelling	1256	0.43	25.8	10.8	42%
	Long (≥ 1 min)	Resting	190	2.68	160.8	67.8	42%
		Foraging	76	2.87	172.2	94.8	55%
		Travelling	88	2.10	126.0	79.2	63%
	All dives	Resting	1140	0.69	41.4	60.6	146%
		Foraging	679	0.60	36.0	58.8	163%
		Travelling	1344	0.54	32.4	33.6	104%
Adult Males	Short (< 1 min)	Resting	1296	0.28	16.8	9.6	57%
		Foraging	1340	0.29	17.4	11.4	66%
		Travelling	1879	0.41	24.6	10.8	44%
	Long (≥ 1 min)	Resting	241	2.59	155.4	45.6	29%
		Foraging	93	3.84	230.4	121.2	53%
		Travelling	106	1.47	88.2	34.8	39%
	All dives	Resting	1140	0.64	38.4	54.0	141%
		Foraging	1433	0.52	31.2	61.8	198%
		Travelling	1985	0.47	28.2	19.2	68%

Mean dive times of juvenile and adult male resident killer whales for dives <1 minute, dives ≥ 1 min, and all dives combined while individuals were resting, foraging and travelling. Also shown are standard deviation, coefficient of variation (CV) and numbers of dives (sample size) per behavioural state.
