# Supplementary material for: Killer whale respiration rates
Source: PLoS One. 2024 May 15;19(5):e0302758. doi: 10.1371/journal.pone.0302758 (PMC11095751; doi:10.1371/journal.pone.0302758)
Supplement: S1 Appendix — (PDF) [file pone.0302758.s002.pdf]

S1 Appendix: Video and TDR data processing and synchronization

Video data collection and processing on drones

We collected video data from a UAV (i.e., drone) from eight of the 11 resident killer whales carrying the animal-borne tags. Once an individual whale was successfully tagged, we deployed a drone to follow the tagged whale and take video footage of the whale at the surface. The drone turned off its video when the tagged whale was no longer visible at the surface during longer duration dives (to conserve battery), or when the drone was taking photographs instead of recording video for concurrent studies. We had occasional instances where the drone battery died midway through observing a focal whale. Consequently, the drone video data collected were not random subsamples of all dives recorded by the animal-borne tags and were biased towards shallower dives. We collected drone video data on some deeper and longer duration dives (up to 58 m), but these dive types did not have the same probability of being captured on the drone video as were shallow dives. We observed each dive from the drone video and recorded the dive duration, surface interval duration, and the behavioural state of the dive.

Time-depth data processing

Time-depth data from the animal-borne dataloggers were zero-offset corrected by calibrating depth data in MATLAB [1]. Animal-borne dataloggers originally sampled at 50 Hz were down-sampled to 2 Hz. All subsequent data processing and analysis were carried out with the statistical software program R 4.1.2 [2].

Dives and surface intervals were defined with a 0.5 m minimum dive threshold on time-depth data at 2 Hz [3]. We selected a shallow minimum dive depth threshold because subsequent analysis focused on capturing all of the respirations at the surface. We tested the different minimum dive thresholds ranging from 0.5 to 2.0 m on a subset of animals and concluded that 0.5 m lined up the best with the surface intervals on the drone by visual analysis. Deeper dive thresholds excluded some surface intervals that were respirations on drone.

Matching dives on animal-borne dataloggers to individual dives on drone video

Additional data processing was required to align the drone and animal-borne tag clocks because they were deployed from different field computers. The time-stamps of the animal-borne tag and drone video were aligned visually by plotting the raw depth vs. time (at 2 Hz) with the dive and surface interval start/end times outputted from the package *diveMove* [3]. Next, we overlaid the start and end times of the dives and surface intervals on the drone. We used unique surface intervals as points of reference (e.g., comparatively longer dives and longer surface interval durations) and drone video field notes to manually calculate an “offset” and correct the drone time-stamps to be accurately synced with the animal-borne tag time-stamps (offsets ranged from 109 to 281 seconds). This process was repeated for all whales and all dives with drone video. Animals that had drone video deployed on the same day should have had the exact same offset because the drone clock and field computer clock was the same. Analysis showed that R48, R58 and D26, D21 had the same offset in pairs calculated independently from each other, indicating that re-alignments were accurate.

The dives and surface intervals identified in the drone videos were matched to corresponding dives on the animal-borne tag in R based on a common time vector of surface interval start/end times synced to both datasets at the level of the individual dive. We used both the start and the end of the surface interval duration to match dives between animal-borne tags and drone videos. Matching the dives using two checkpoints ensured that the alignment was accurate. Accuracy of matching was verified by visual plots for each animal for all dives.

References

1. Cade DE, Gough WT, Czapanskiy MF, Fahlbusch JA, Kahane-Rapport SR, Linsky JM, et al. Tools for integrating inertial sensor data with video bio-loggers, including estimation of animal orientation, motion, and position. Animal Biotelemetry. 2021;9(34):1-21. doi: 10.1186/s40317-021-00256-w.
2. R Core Team. R: A language and environment for statistical computing. Vienna, Austria: R Foundation for Statistical Computing; 2022.
3. Luque SP. Diving behaviour analysis in R. R News. 2007;7(3):8-14.
