# Supplementary material for: Killer whale respiration rates
Source: PLoS One. 2024 May 15;19(5):e0302758. doi: 10.1371/journal.pone.0302758 (PMC11095751; doi:10.1371/journal.pone.0302758)
Supplement: S2 Appendix — (PDF) [file pone.0302758.s003.pdf]

# S2 Appendix: Hierarchical hidden Markov model details

## Summary

The following supporting information details the characteristics of individual dives of killer whales recorded on drone video, as well as the procedures used to build the hierarchical hidden Markov model (HHMM) used to predict the behaviours of killer whales from time-depth recorders without any information from drone video (unknown dives).

## Individual dive characteristics from drone video used to build HHMM

Our use of an HHMM was motivated by the fact that only a subset of dives had drone video matched to the animal-borne tag data and that HHMMs have been successful in predicting behaviours from dive data [1].

To build our HHMM, we first examined how representative the dives on the drone footage were relative to common dive characteristics of killer whales. Table A represents the dive characteristics of the individual dives that had drone video matched to animal-borne dataloggers.

As expected, logging behaviour had shallower dive depths than foraging or travelling for both adult male and juvenile killer whales. Foraging had an extremely high standard deviation in dive duration and maximum dive depth relative to the other behavioural states of juveniles. The standard deviations in dive duration and maximum dive depth attained by juveniles were more than twice the mean values for these dive characteristics (Table A). This indicates that foraging was less stereotypical overall relative to the other behavioural states examined in juveniles. Adult males had deeper maximum dive depths and longer dive durations while travelling than while foraging. Foraging dives of adult males had lower standard deviations relative to the same behaviours in juveniles. However, our sample sizes of dives with drone video were smaller for adult males, and we recorded limited logging or travelling behavioural states relative to resting and foraging.

## Details for building Hidden Markov Models to predict the behaviour of a track of dives

The HHMM was fit using individual killer whale dives as fine-scale observations. We assumed that there was a total of four (fine-scale) dive types: shallow (< 7.5 m), medium (10-30 m), deep (> 50 m) and logging. The first three dive types were selected from visual inspection of dive profiles as well as histograms of maximum depth (Fig A). Logging behaviour was unique in that it was characterized by much longer surface intervals than the other three dive types. Logging behaviour was rarely recorded by the drone and did not occur for tracks >10 minutes in duration; therefore, logging was treated as a dive type in the HHMM rather than as a behavioural state predicted by the HHMM. We summarized each killer whale dive with three dive characteristics (maximum depth in meters, dive duration in seconds, and surface interval duration in seconds) from the animal-borne tags. Each characteristic was assumed to follow a gamma distribution that was a function of the underlying dive type. All three characteristics were assumed to be independent of one another after conditioning on its corresponding underlying dive type and the behavioural state of its associated track (see below).

For the coarse scale of the HHMM, each time series of dives was subdivided into approximately 10-minute long “tracks” [tracks in current study are equivalent to segments in 1].

The tracks were not exactly 10 minutes because we wanted to ensure that they started at the beginning of a dive and ended at the end of a surface interval. As such, we defined a track as the shortest continuous sequence of dives and surface intervals that was ≥ 10 minutes in cumulative duration (sum of all the individual dives and surface intervals). If a track reached the 10-minute threshold while at depth, the track terminated at the end of the next complete surface interval (i.e. a track could not end in the middle of a dive at depth or in the middle of a surface interval and ended at the end of the following surface interval).This definition ensured that coarse-scale behavioural state lasted for at least 10 minutes and consisted of full dives and complete surface intervals. This definition does mean that not all surface intervals associated with recovery after a deeper dive are included in the same track as the deeper dive (see the respiration rates section of the discussion for more details). We selected a total of three behavioural states (resting, travelling, and foraging), which is in line with the behaviours observed via drone footage and previous studies on killer whales [2].

We assumed that the distribution of each dive characteristic depended upon the underlying dive type (shallow, medium, deep, or logging), but not on the underlying behaviour of the track (resting, travelling, or foraging). This choice is in line with Leos-Barajas et al. [1], and we found that the HHMM produced more biologically meaningful results when the distributions of the dive characteristics were shared across behavioural states of tracks. Namely, when the distributions of dive characteristics were allowed to vary among behavioural states, the HHMM produced behavioural states that did not clearly correspond to resting, travelling, and foraging.

We assigned labels to the behavioural states of tracks using drone videos. While our model assigned behaviours to tracks, not individual dives, we originally used drone videos to identify the behaviour of individual dives in order to label track behaviour. As such, we assigned the behaviour of drone-identified dives to the tracks that contained them (i.e., these tracks had observed labels, not unobserved or predicted). In the few cases where tracks contained dives with conflicting drone-identified behaviours, we followed the following selection scheme: 1) if a track contained both a drone-identified foraging dive and either a travelling or resting drone-identified dive, then that track was labelled as foraging; and 2) if a track contained both a drone-identified travelling and a resting dive, but no drone-identified foraging dive, then no labels were assigned to the track and the HHMM predicted the behaviour of the track. This scheme prevented us from assigning contradictory labels to tracks and prioritized the identification of foraging behaviour. We incorporated all labels of track behaviour using a method similar to that of Li et al. [3].

One subset of dives had incomplete drone video footage and we had reason to believe that the dives were foraging dives based on field observations. When an animal did a longer duration dive, the drone often lost sight of where the whale would surface and did not capture the full post-dive surface interval of that dive. We assumed that these specific dives were foraging because video indicated that they were longer in duration, often had fluking present, and the animal-borne tag data confirmed deeper depths compared to synchronized dives. In addition, since research has shown that dives > 30 m are generally linked with foraging [4], our HHMM forced tracks containing a dive > 30 m to be foraging by 1) fixing the probability of starting a non-foraging track in a deep dive as zero; and 2) fixing the probability of jumping from any other dive to a "deep" dive as zero during non-foraging tracks. Further, if any dive was deeper than 30 m and also within 2 minutes of a dive that was drone-identified as foraging, that dive was labelled as "deep" (even if it was shallower than 50 m) and the track associated with it was labelled as foraging. This assumed that

two deep dives close together in time were both foraging if one of them was identified as such by drone video.

The majority of dives were shallow dives (<7.5 m), so a standard HHMM without individual dive-level labels may have assigned multiple dive types to these shallow dives while treating deep dives as outliers. We were interested in deep foraging dives [4] as well as distinct medium-depth dives, so we created individual dive-level labels to ensure that the HHMM identified these dive types. Namely, we visually inspected the data and made dive type labels informed by the previous literature on killer whale behaviour. Specifically, we determined labels of dive type using the maximum dive depth and post-dive surface interval duration according to the following criteria:

1. Dives with post-dive surface intervals ≥ 10 seconds were labeled as "logging" behavioural states and logging dive types. This threshold was used because all video-identified logging dives had post-dive intervals of ≥ 10 seconds, and all other video-labelled dives had post-dive surface intervals < 10 seconds.
2. Dives < 7.5 meters were labelled as "shallow". This threshold was determined via visual inspection of a histogram of maximum depths (Fig A). If a dive was > 7.5 m and < 10 m, the HHMM model assigned the depth category without explicitly being defined as either shallow or medium.
3. Dives between 10 meters and 30 meters were labelled as "medium". If a dive was > 30 m and < 50 m, the HHMM model assigned the depth category without explicitly definition as either medium or deep. The 10 m threshold was determined via visual inspection (Fig A), and the 30 m threshold was determined because previous studies have concluded that foraging does not occur at depths shallower than 30 meters [4].
4. Dives > 50 meters were labelled as "deep". Other studies have found that dives > 30 meters are more often linked with foraging; however, we chose the deep threshold of 50 meters due to visual inspection of a histogram of maximum depths (Fig A) and to be more conservative. This allowed the results to be more biologically interpretable as the HHMM assigned the depth category as either medium or deep if the depth was between 30 meters and 50 meters.

To account for behavioural differences among sexes and age classes, it would be ideal to fit separate HHMMs for adult male, adult female, and juvenile killer whales. Age classes were defined as juveniles 4-12 years, adult males > 13 years, and adult females > 12 years and a recorded birth [5-7]. However, our data set includes only one confirmed adult female killer whale over the age of 12 years (R48, who was 14 years old) [8]. For our study, we grouped the one young adult female into the juvenile category as it is not possible to do an HHMM, cross-validation, or compare respiration rates using an LME model with only 1 animal in this group. Therefore, we included R48 with the juvenile killer whales to form a "juvenile" HHMM. R48 is a very young adult female and female killer whales can reach maturity anywhere between 12-17 years of age [5]. Based on R48's age and size, it's reasonable that her behaviour and physiology is still similar to the other juveniles in the HHMM. For example, the dive profiles of R48 and R58 were similar. To determine if R48 impacted the juvenile HHMM, we fit 2 models (with and without R48 included) and compared the results. We compared the output from both models to see if they differed in which behavioural state was predicted for all tracks. Even if R48 is removed, the 2 models agree in the predicted behavioural state 97% of the time (n=169 total tracks).

All HHMM analyses were done using the *momentuHMM* package in R [9]. Because maximum dive depth, dive duration, and post-dive surface intervals were used as dive-level

emissions within the HHMM, we did not perform any post-hoc tests to determine if dive characteristics vary significantly between dive types. We found that the model produced biologically meaningful results, and we validated the accuracy of the model using cross validation (Table 3, Table 5). Future studies may incorporate the dive type thresholds above by using truncated gamma distributions to model maximum depth and post-dive surface intervals. However, truncated gamma distributions were not accessible in the *momentuHMM* package [9].

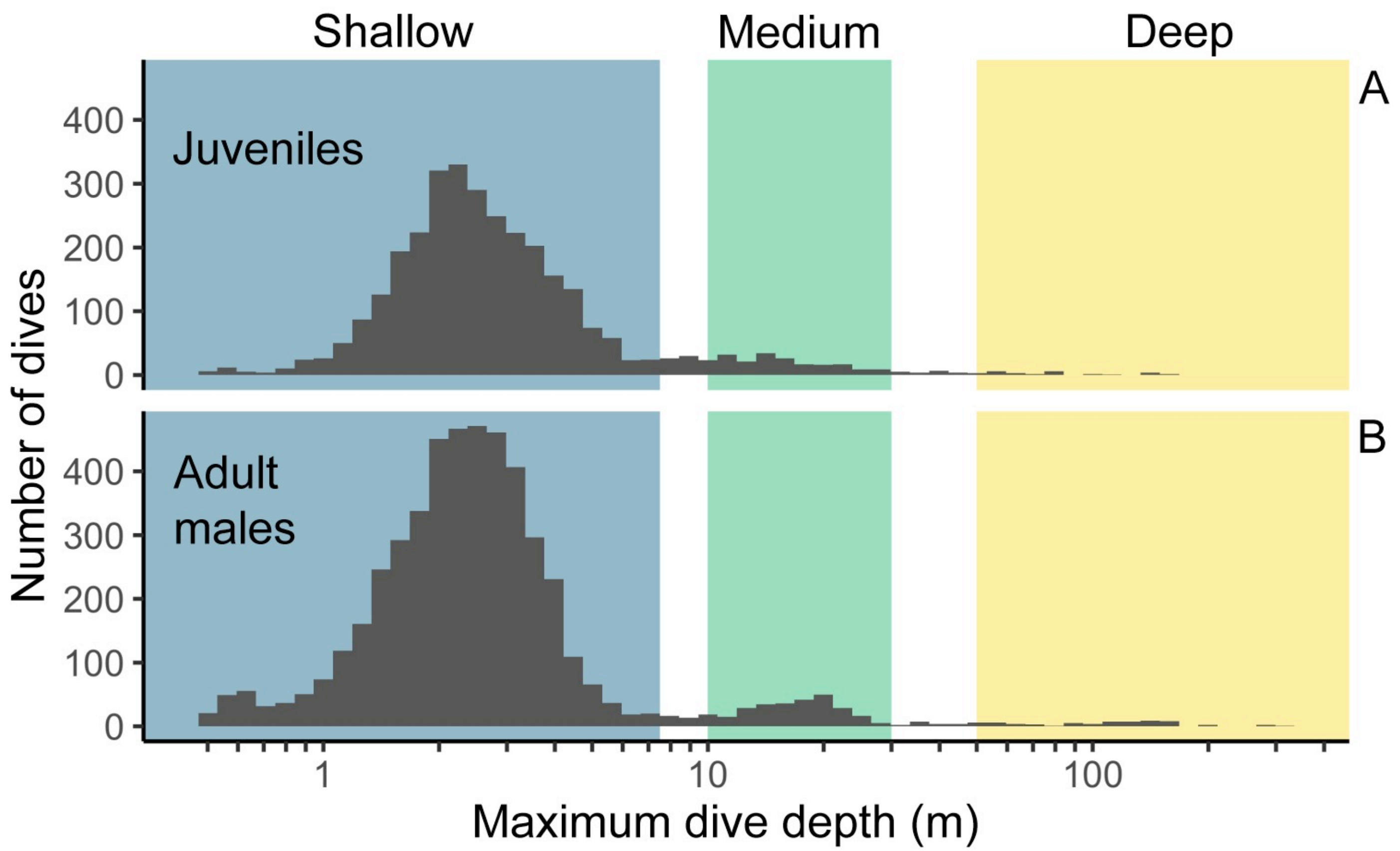


**Fig A. Histogram of maximum dive depths for HHMM dive types.** Dive depths are plotted on a log scale for 4 adult male (n=4955 dives) and 7 juvenile resident killer whales (n=3163 dives). Note that this is the same as Fig 2 in the manuscript.

**Table A. Dive characteristics of eight northern resident killer whales recorded by animal-borne tags and categorized by behavioural state observed on drone videos.**

| | Behavioural state | Dives | Dive duration (min) | | | Surface interval (min) | | | Max dive depth (m) | | |
|---|---|---|---|---|---|---|---|---|---|---|---|
| | | N | Mean | S.D. | Range | Mean | S.D. | Range | Mean | S.D. | Range |
| **Juveniles** | Logging | 7 | 0.33 | 0.35 | 0.08-1.10 | 0.52 | 0.46 | 0.05-1.41 | 1.78 | 1.03 | 0.89-3.43 |
| | Resting | 50 | 0.35 | 0.42 | 0.08-2.38 | 0.06 | 0.01 | 0.03-0.08 | 2.34 | 2.02 | 1.10-12.20 |
| | Foraging | 28 | 0.53 | 0.95 | 0.09-4.97 | 0.04 | 0.01 | 0.03 - 0.05 | 8.15 | 14.07 | 1.50-57.93 |
| | Travelling | 304 | 0.46 | 0.44 | 0.01 – 4.67 | 0.04 | 0.01 | 0.02 - 0.08 | 2.51 | 2.00 | 0.55-26.13 |
| **Adult Males** | Logging | 1 | 0.18 | NA | NA | 1.73 | NA | NA | 1.13 | NA | NA |
| | Resting | 33 | 0.40 | 0.54 | 0.05-2.42 | 0.06 | 0.01 | 0.04-0.08 | 2.73 | 2.58 | 0.79-12.30 |
| | Foraging | 51 | 0.32 | 0.14 | 0.02-0.61 | 0.06 | 0.02 | 0.03-0.13 | 2.12 | 0.61 | 0.59-4.17 |
| | Travelling | 2 | 0.50 | 0.15 | 0.39-0.60 | 0.05[a] | NA | NA | 4.19 | 1.68 | 3.00-5.37 |

Behavioural states observed on drone videos, sample size of individual dives per behavioural sate on drone (N), and summary statistics for dive durations, surface interval durations, and maximum depths from dives that had overlapping drone video and animal-borne dataloggers. Behavioural states are defined in Table 2. Sample size of whales with drone video for all behaviours was n=6 juveniles (389 dives) and n=2 adult males (87 dives).

[a] For adult males travelling behavioural state, both individual surface intervals were 0.05 minutes.

# References

1. Leos-Barajas V, Gangloff EJ, Adam T, Langrock R, Van Beest FM, Nabe-Nielsen J, et al. Multi-scale modeling of animal movement and general behavior data using hidden Markov models with hierarchical structures. Journal of Agricultural, Biological and Environmental Statistics. 2017;22:232-48. doi: 10.1007/s13253-017-0282-9.
2. Noren DP, Hauser DD. Surface-based observations can be used to assess behavior and fine-scale habitat use by an endangered killer whale (*Orcinus orca*) population. Aquatic Mammals. 2016;42(2). doi: 10.1578/AM.42.2.2016.168.
3. Li J, Lee J-Y, Liao L. A new algorithm to train hidden Markov models for biological sequences with partial labels. BMC Bioinformatics. 2021;22:1-21. doi: 10.1186/s12859-021-04080-0.
4. Wright BM, Ford JK, Ellis GM, Deecke VB, Shapiro AD, Battaile BC, et al. Fine-scale foraging movements by fish-eating killer whales (*Orcinus orca*) relate to the vertical distributions and escape responses of salmonid prey (*Oncorhynchus spp.*). Movement Ecology. 2017;5(3):1-18. doi: 10.1186/s40462-017-0094-0.
5. Olesiuk PF, Ellis GM, Ford JK. Life history and population dynamics of northern resident killer whales (*Orcinus orca*) in British Columbia. Nanaimo, British Columiba: 2005 Contract No.: 2005/045.
6. Noren DP. Estimated field metabolic rates and prey requirements of resident killer whales. Marine Mammal Science. 2011;27(1):60-77. doi: 10.1111/j.1748-7692.2010.00386.x.
7. DFO. Population status update for the northern resident killer whale (*Orcinus ocra*) in 2020. Ottawa, ON: 2021.
8. Towers JR, Pikington JF, Gisborne B, Wright BM, Ellis G, Ford J, et al. Photo-identification catalogue and status of the northern resident killer whale population in 2019. Nanaimo, BC: 2020 3371.
9. McClintock BT, Michelot T. momentuHMM: R package for generalized hidden Markov models of animal movement. Methods in Ecology and Evolution. 2018;9(6):1518-30. doi: 10.1111/2041-210X.12995.
