# Supplementary material for: Killer whale respiration rates
Source: PLoS One. 2024 May 15;19(5):e0302758. doi: 10.1371/journal.pone.0302758 (PMC11095751; doi:10.1371/journal.pone.0302758)
Supplement: S3 Appendix — (PDF) [file pone.0302758.s004.pdf]

S4 Appendix: Mass-specific oxygen consumption (VO_2) calculations

Summary

This supporting information provides details for VO_2 calculations including estimates of predicted body length, predicted body mass, tidal volume (V_T), oxygen extraction from inhaled air ($E_{O2,\%}$), and oxygen uptake per respiration (T_{O2}, L O_2 breath^{-1}) per individual animal that were used to estimate VO_2 in Table 6 of the full manuscript.

Our goal was to predict mass-specific V_T to calculate VO_2, but we could not accurately measure body mass or length from the drone video because this requires several images (~5 images per whale) for reliable estimates [1]. However, all animals were of known age from photo-identification. Therefore, we developed a sex-specific Gompertz growth model predicting length-at-age from the entire 2020 population, and used this estimated length (cm) to estimate body mass (kg) for each of the 11 study whales from a pre-existing length-mass model [2]. Lengths of juvenile animals were estimated using the female growth curve. We then matched the 11 tagged whales in our study to "proxy whales" in Kriete [Table 16 in 3] that had similar body masses per sex and also similar age classes based on [4-6]. These "proxy whales" also had reported mass-specific V_T per whale [Table 1 in 3]. These mass-specific V_T were subsequently used to estimate VO_2 per whale, per track, and per behaviour. Notably, a mass-specific V_T also yields mass-specific E_{O2} and T_{O2} estimates per sex and per behavioural state.

Prediction of body length and body mass from age

Body length (m) was predicted from age as of 2020 using a Gompertz growth model per sex based on published data [n=23, Table 1 in 1]. To clarify, we derived the Gompertz growth model based on n=43 southern resident killer whales in Fearnbach et al. [1], then applied the resulting model growth curve to the 11 whales tagged in 2020 with different equations per sex. For the growth curves, we assumed that animals of unknown sex in our dataset were females. We believe this assumption is reasonable because immature male body proportions are more similar to females in length and body mass than to those of larger adult males. Both males and female killer whales follow a similar growth curve until they reach 11-15 years of age, at which time males dramatically increase in size [4]. We then used the estimates of body length of the study animals to predict their body mass (kg) based upon the length-mass equation from Bigg and Wolman [2, note that this equation is not-sex specific].

The killer whales in our study were then matched to whales (termed "proxy whales") that had similar body masses per sex [Table 16 in 3]. Kriete [3] presents data on both body mass (Table 16) and tidal volume (Table 1) for 4 whales for 4 different activity states. As tidal volume is affected by body size, we calculated mass-specific tidal volume for each of the whales in Kriete [3]. Given that body mass was predicted from length and age information, proxy whales also matched animals in the current study in both age class and sex. For the same reason, we only calculated VO_2 (which was based on tidal volume) for animals that had predicted body masses that were reasonably matched within 15% of the body masses in Kriete [Table 16 in 3]. Animals A113, I129, and I145 were excluded from VO_2 calculations because their predicted body masses were more than 15% different (364-754 kg difference) than the predicted body masses and V_T of the female killer whales in Kriete [Table 16 in 3]. The predicted body masses for all 4 adult males

in our study ranged from 4172 to 3382 kg, but only males L87 and L88 were within 15% of the predicted body masses available in Kriete [males were 2,800 or 4,733 kg in 3]. Whale I129 was excluded because its predicted body mass was not within 15% of either proxy male whale in Kriete [3, Table A in S4 Appendix].

Calculating mass-specific tidal volumes per animal

Other studies [5, 7] have used a fixed tidal volume per sex based on the max V_T reported in Kriete [3], specifically 149 L for females and 258.5 L for larger males. For our VO_2, we chose the maximum tidal volume of the proxy animal measured in Kriete [3] with the appropriate activity state in Table 1 [3]. It is important to note that the body masses in Kriete [3] were not measured directly, but were estimated from measured body lengths [Table 16 in 3] using the same length-mass equation as in our study [2].

Mass-specific V_T for the study whales while in a resting behavioural state was from the tidal volume reported during "Activity level 1" of the matched "proxy whale" [Table 9 in 3]. Activity level 1 was measured while trained killer whales rested. For foraging and travelling behavioural states of the study animals, we used V_T, E_{O2}, and T_{O2} from activity level 2 in Kriete [3]. Activity level 2 was measured from trained animals undertaking light to moderate swimming and shallow diving activities. Tidal volume for activity level 2 was measured on females, but not on the males in Kriete [3]. For males, we averaged maximum mass-specific V_T from activity level 1 and 3 from activity level 2 for foraging and travelling [Table 1 in 3]. The larger whale in Kriete [3] was the only male whale that matched our tagged male whales within 15%.

Calculating VO_2

The calculations for VO_2 was based on a tidal volume that was mass-specific per animal per activity level 1 or activity level 2 [VT = Table 1, body mass = Table 16 in 3]. A mass-specific V_T resulted in mass-specific E_{O2} and T_{O2} estimates as well. Our calculations of VO_2 and Table S4 below only includes tracks that were ≥ 10 min cumulative duration.

As with V_T, mean oxygen extraction from inhaled air (E_{O2}, %) and oxygen uptake per respiration (T_{O2}, L respiration^{-1}) also varied per sex, per age-class, per behaviour, and per individual animal on a mass-specific basis. Rather than apply the same E_{O2} to all behaviours, we used the E_{O2} values that best matched the behavioural state definitions in our study. As such, resting E_{O2} came from trained killer whales while they rested (activity level 1), and foraging and travelling were estimated from trained animals undertaking light to moderate swimming and shallow diving activities [activity level 2, Table 9 in 3]. For males only, E_{O2} for activity level 2 was averaged from activity levels 1 and 3 values for both whales because it was not directly measured to yield a single mean E_{O2} for all males in activity level 2.Within the males, only whales L88 and L87 were included because the predicted body masses of I107 and D21 differed by > 15% from the male whale in Kriete [3]. The number of total tracks per behaviour are noted in Table 6. The number of animals per sex per behaviour varies depending on which animals exhibited each behavioural state (n=4 females, n=3 males across all behaviours). There was reasonable distribution of the animals across all behaviours except for foraging males. Foraging males VO_2 included only animal L87 because L88 did not have any foraging tracks. See Table 6 for the VO_2 estimates per behaviour.

Table A. Predicted body lengths, body masses, and calculated oxygen consumption for 11 northern and southern resident killer whales derived from age as of July 2020.

Whale ID	Age Class & Sex [a]	Age (yr)	Predicted body length (m) [b]	Predicted body mass (kg) [c]	Predicted Body mass and age (kg, years) of proxy whale to estimate V_T [d]	Absolute body mass difference with proxy whale (kg, % difference)	Mass-specific maximum V_T (L) [e]	Activity state in Kriete (1995) [f]	Behavioural state in current study	E_{O2} [g] (%)	T_{O2} [h] (%)
R58	Juvenile (unknown)	9	5.3	2137	2005, 11, (Vigga)	132 (+7%)	42.0	Level 1	Resting	35.1	3.08
							55.0	Level 2	Travelling	40.1	4.61
							55.0	Level 2	Foraging	40.1	4.61
A100	Juvenile (unknown)	9	5.3	2137	2005, 11, (Vigga)	132 (+7%)	42.0	Level 1	Resting	35.1	3.08
							55.0	Level 2	Travelling	40.1	4.61
							55.0	Level 2	Foraging	40.1	4.61
D26	Juvenile (unknown)	10	5.4	2272	2005, 11, (Vigga)	267 (+13%)	42.0	Level 1	Resting	35.1	3.08
							55.0	Level 2	Travelling	40.1	4.61
							55.0	Level 2	Foraging	40.1	4.61
R48	Female (adult)	14	5.7	2666	2800, 21, (Yaka)	134 (-5%)	149.0	Level 1	Resting	35.1	10.96
							114.0	Level 2	Travelling	40.1	9.57
							114.0	Level 2	Foraging	40.1	9.57
I129	Juvenile (unknown)	11	5.5	2391	2005, 11, (Vigga)	386 (+20%) *excluded*	NA	NA	NA	NA	NA
I145	Juvenile (unknown)	6	4.7	1641	2005, 11, (Vigga)	364 (-19%) *excluded*	NA	NA	NA	NA	NA
A113	Juvenile (Female)*	4	4.3	1252	2005, 11, (Vigga)	754 (-38%) *excluded*	NA	NA	NA	NA	NA
L87	Adult (male)	28	6.8	4172	4733,20 (Hyak)	561 (-12%)	254.5	Level 1	Resting	35.2	18.77
							256.5	Level 2	Travelling	41.2	22.11
							256.5	Level 2	Foraging	41.2	22.11
L88	Adult (male)	27	6.8	4148	4733,20 (Hyak)	585 (-12%)	254.5	Level 1	Resting	35.2	18.77
							256.5	Level 2	Travelling	41.2	22.11
							256.5	Level 2	Foraging	41.2	22.11
I107	Adult (male)	16	6.4	3499	2800, 11 (Finna)	699 (+25%) *excluded*	NA	NA	NA	NA	NA
D21	Adult (male)	15	6.3	3382	2800, 11 (Finna)	582 (+21%) *excluded*	NA	NA	NA	NA	NA

[a] Sex of unknown young animals were treated as females for morphometrics and growth curves.

*Photo identification confirmed sex [8]

[b] Body length (m) was predicted from age using a Gompertz growth model per sex on published data of southern resident killer whales [Table 1 in 1].

[c] Body mass (kg) was predicted from body length (m converted to cm) using Bigg and Wolman [2].

[d] Whales in our study were matched to whales that had similar body masses per sex using the whale's most recent estimated mass [Table 16 in 3]. Animals were also matched by age class which was a function of mass. Animals A113, I129, I145, I107, and D21 were excluded from VO_2 calculations because their predicted body masses were not within 15% of the predicted body masses and V_T of the killer whales in Kriete [Table 16 in 3].

[e] Maximum mass-specific tidal volumes (V_T) were matched based on similar body masses per activity level per sex from Kriete [Table 9 and Table 1 in 3]. V_T for activity level 2 on the males was not measured directly in Kriete [3]. For males only, we averaged maximum mass-specific V_T from activity level 1 and 3 from Hyak for activity level 2 for foraging and travelling.

[f] Activity level 1 was measured while trained killer whales rested. Activity level 2 was measured from trained animals undertaking light to moderate swimming and shallow diving activities.

[g] Mean oxygen extraction from inhaled air (E_{O2}, %) for resting was from activity level 1, and foraging and travelling was from activity level 2 per sex [Table 9 in 3]. For males only, E_{O2} for activity level 2 was averaged from activity levels 1 and 3 values for Hyak because it was not directly measured in Kriete [3]. For all calculations, we assumed the proportion of oxygen in air was 0.2095.

[h] Oxygen uptake per respiration (T_{O2}, L O_2 breath^{-1}) varies per animal by body mass and by activity state because it is based on mass-specific V_T.

References

1. Fearnbach H, Durban JW, Ellifrit DK, Balcomb III KC. Size and long-term growth trends of endangered fish-eating killer whales. Endangered Species Research. 2011;13(3):173-80. doi: 10.3354/esr00330.
2. Bigg MA, Wolman AA. Live-capture killer whale (*Orcinus orca*) fishery, British Columbia and Washington, 1962–73. Journal of the Fisheries Board of Canada. 1975;32(7):1213-21. doi: 10.1139/f75-140.
3. Kriete B. Bioenergetics in the killer whale, *Orcinus orca*: University of British Columbia; 1995.
4. Olesiuk PF, Ellis GM, Ford JK. Life history and population dynamics of northern resident killer whales (*Orcinus orca*) in British Columbia. Nanaimo, British Columiba: 2005 Contract No.: 2005/045.
5. Noren DP. Estimated field metabolic rates and prey requirements of resident killer whales. Marine Mammal Science. 2011;27(1):60-77. doi: 10.1111/j.1748-7692.2010.00386.x.
6. DFO. Population status update for the northern resident killer whale (*Orcinus ocra*) in 2020. Ottawa, ON: 2021.
7. Roos MM, Wu G-M, Miller PJ. The significance of respiration timing in the energetics estimates of free-ranging killer whales (*Orcinus orca*). Journal of Experimental Biology. 2016;219(13):2066-77. doi: 10.1242/jeb.137513.
8. Towers JR, Pikington JF, Gisborne B, Wright BM, Ellis G, Ford J, et al. Photo-identification catalogue and status of the northern resident killer whale population in 2019. Nanaimo, BC: 2020 3371.
